# A framework for the biophysical screening of antibody mutations targeting solvent-accessible hydrophobic and electrostatic patches for enhanced viscosity profiles

**DOI:** 10.1016/j.csbj.2024.05.041

**Published:** 2024-05-24

**Authors:** Georgina B. Armstrong, Vidhi Shah, Paula Sanches, Mitul Patel, Ricky Casey, Craig Jamieson, Glenn A. Burley, William Lewis, Zahra Rattray

**Affiliations:** a Drug Substance Development, GlaxoSmithKline, Gunnels Wood Road, Stevenage, UK; b Large Molecule Discovery, GlaxoSmithKline, Gunnels Wood Road, Stevenage, UK; c Department of Pure and Applied Chemistry, University of Strathclyde, Glasgow, UK; d Strathclyde Institute of Pharmacy and Biomedical Sciences, University of Strathclyde, Glasgow, UK

**Keywords:** Antibody, Viscosity, Physicochemical descriptors, Developability, Protein engineering

## Abstract

The formulation of high-concentration monoclonal antibody (mAb) solutions in low dose volumes for autoinjector devices poses challenges in manufacturability and patient administration due to elevated solution viscosity. Often many therapeutically potent mAbs are discovered, but their commercial development is stalled by unfavourable developability challenges. In this work, we present a systematic experimental framework for the computational screening of molecular descriptors to guide the design of 24 mutants with modified viscosity profiles accompanied by experimental evaluation. Our experimental observations using a model anti-IL8 mAb and eight engineered mutant variants reveal that viscosity reduction is influenced by the location of hydrophobic interactions, while targeting positively charged patches significantly increases viscosity in comparison to wild-type anti-IL-8 mAb. We conclude that most predicted *in silico* physicochemical properties exhibit poor correlation with measured experimental parameters for antibodies with suboptimal developability characteristics, emphasizing the need for comprehensive case-by-case evaluation of mAbs. This framework combining molecular design and triage via computational predictions with experimental evaluation aids the agile and rational design of mAbs with tailored solution viscosities, ensuring improved manufacturability and patient convenience in self-administration scenarios.

## Highlights

• We present computational design of a mutant panel, targeting hydrophobic or charged solvent-accessible surface patches.
• Computational and experimental biophysical approaches were used in combination to engineer and evaluate mutant variants.
• Cross-correlations showed site-dependent reduction in hydrophobicity lowered viscosity at high antibody concentration.

## Graphical Abstract

**Figure:**
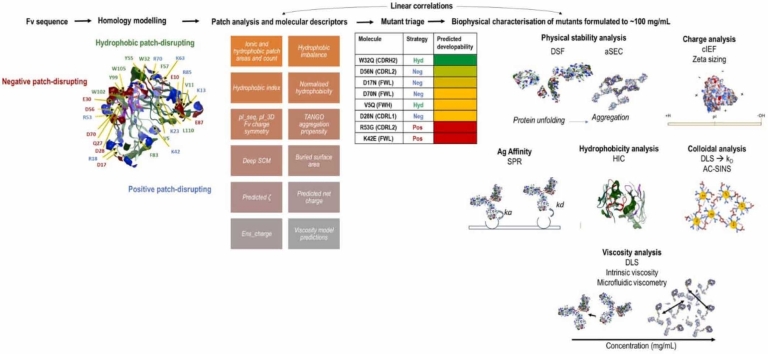

## 1 Introduction

Therapeutic monoclonal antibodies (mAbs) have emerged as indispensable tools in the treatment of chronic diseases such as diabetes, cancer, and autoimmune disorders. [1] Empowering patients with self-administration regimens, subcutaneous injection is the route of administration of choice for the delivery of these life-changing therapies, necessitating formulation design strategies to accommodate small injection volumes. [2] However, this pursuit of patient convenience presents a formidable challenge: how to achieve high mAb formulation concentrations (>100 mg/mL) at low dose volumes (0.5–1 mL) without facing developability challenges, a term which refers to the likelihood of a mAb molecule to become a suitable candidate in the context of manufacturability, safety, and efficacy at a reasonable cost and timeframe. [3] Developability challenges in the context of mAbs include a high risk of aggregation and elevated solution viscosity at dose-relevant concentrations, which have implications for quality, safety and efficacy throughout the mAb product lifecycle. [4]

The viscosity of mAb formulations, a critical parameter governing dosing and delivery efficacy, is intricately linked to protein-protein interactions arising from the mAb amino acid sequence and formulation excipient composition. [5], [6]

High concentration mAb formulations exacerbate these interactions, leading to increased aggregation risk and elevated formulation viscosity (>30 centipoise). [7] High mAb formulation concentrations result in an exponential increase in protein-protein interactions leading to a higher aggregation risk. The diffusion interaction parameter (k_D_) is used to measure protein-protein interactions and colloid stability, with high viscosity mAbs generally exhibiting large negative k_D_ values (attractive). [8], [9], [10]

In this pursuit, various strategies have been employed to modulate protein-protein interactions and mitigate mAb solution viscosity. These strategies have ranged from the alteration of electrostatic properties by changing formulation buffer pH and salt composition, to employing viscosity reducing excipients (e.g., amino acids) to increase the solubility of partially folded and unfolded states. [11], [12], [13] Furthermore, advancements in sequence-based engineering offer a promising avenue for targeting solvent-accessible electrostatic patches on the mAb surface, with the potential to revolutionize the mAb design landscape.

In the emerging era of precision medicine, the integration of high throughput *in silico* predictions and molecular triaging approaches holds immense potential in streamlining early-stage discovery campaigns. [14], [15], [16] By elucidating the intricate relationship between mAb molecular descriptors and developability risks, [17] these cutting-edge approaches empower researchers to more expediently identify candidate mAbs with superior physicochemical properties, paving the way for more agile drug development pipelines with less attrition.

Current landscape analyses and models defining optimal developability for mAbs are based on clinically approved drug products with optimal characteristics. However, amidst these advancements, it is imperative to broaden our focus beyond clinically approved mAbs and encompass those with unknown or sub-optimal developability characteristics. In doing so, we expand our understanding of how to navigate high formulation concentration solution viscosity more effectively, ultimately enhancing the success rate of mAb drug development endeavors. [8]

In this work, we harness computational molecular descriptors as a guiding tool for the design and triage of a mutant mAb panel altering solvent-accessible hydrophobic and electrostatic surface patch area coverage. Through a combined computational and experimental pipeline, we examine the relationship between single-point mutations and the biophysical properties of a model antibody, anti-IL-8 mAb. Our findings show the site-specific and strategy-dependent impact of mutations based on surface patch composition, offering an insight into downstream effects of molecular alterations. We report significant alterations in surface potential from single-point mutations in the variable region and observe favourable developability characteristics for hydrophobic or negative patch-disrupting mutants. We observe correlations between hydrophobicity-based molecular descriptors and colloidal parameters in predicting hydrophobicity-driven self-associations, impacting solution viscosity at high mAb concentrations.

## 2 Results

The goal of this project was to engineer new mutant variants by predicting mutations by modulating antibody electrostatic and hydrophobic properties that control mAb conformation and viscosity.

### 2.1 Generation of the anti-IL-8 mAb mutant panel

Using homology models of anti-IL-8 mAb, we compared the impact of targeting solvent-accessible hydrophobic and charged patches on solution viscosity at high mAb concentration. [18], [19], [20] Patch analysis of a anti-IL-8 mAb wild-type (WT) IgG1 homology model identified residues contributing to positive, negative, and hydrophobic patches as potential candidates for mutation. We then determined mutant physicochemical molecular descriptors and performed patch analysis.

### 2.2 Homology modelling and patch analysis of WT anti-IL-8 mAb

**Fig. 1:**
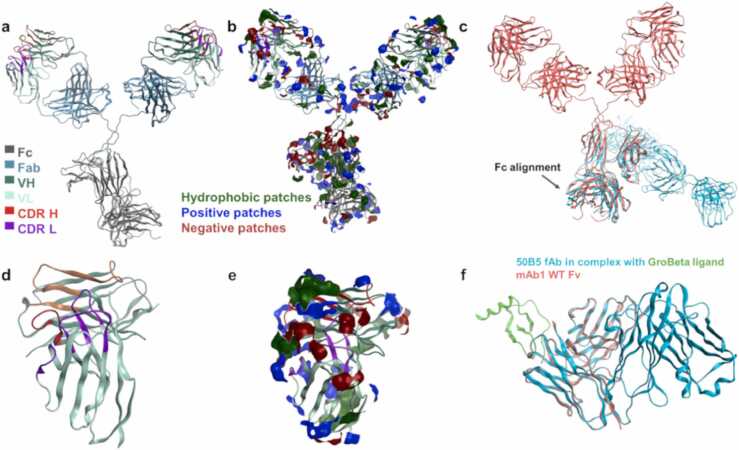
***Homology models of anti-IL-8 mAb.*****a,** the full IgG structure was modelled using the PDB 5OB5 template for the Fab region and IgG model in the MOE platform The Fc (grey), constant light chain 1 and heavy chain 1 (blue), variable heavy chain (dark green) variable light chain (light green), heavy chain CDRs (red) and light chain CDRs (purple) were labelled using Kabat annotation. **b,** the hydrophobic (green), positive (blue), and negative (red) patches applied onto the full IgG1 homology model to demonstrate the exposed surface charges and accessible non-polar regions as potential sites for promoting protein-protein interactions. **c,** superimposition, and alignment of the anti-IL-8 mAb WT full IgG homology model (pink) onto the template 1IGY PDB IgG1 structure (blue) to model the Fc structure. **d,** the Fv region was modelled separately and used for most molecular descriptor calculations. **e,** patch analysis of the Fv to aid identification of candidate sites for single-point mutation. **f,** superimposition and alignment of the anti-IL-8 mAb WT Fv homology construct (pink) onto the template 5OB5 PDB fAb structure (blue) that was in complex with the GroBeta ligand (green).

We constructed homology models of the full anti-IL-8 mAb structure and the variable fragment (Fv) of WT anti-IL-8 mAb in the MOE molecular modelling suite. [21] Since the fragment antigen-binding region (Fab) crystal structure (PDB 5OB5) matched the framework and complementarity determining regions (CDRs) perfectly, patch analysis was conducted on resulting structures (Fig. 1**a**). The surface potential mapped onto the anti-IL-8 mAb surface (Fig. 1**b**), shows negative, positive, and hydrophobic patch distributions.

Overall, we observed the largest contribution to the surface potential of WT anti-IL-8 mAb IgG (Fig. 1**b**) from hydrophobic (3790 Å^2^), positive (2940 Å^2^) and negative (2250 Å^2^) patches, with a net charge of + 22.68 C (pH 6). A similar trend was seen with the anti-IL-8 mAb Fv (Fig. 1**d and e**), with surface area coverage of 520, 160, and 50 Å^2^ for hydrophobic, positive and negative patches, respectively, and a net charge of + 0.05 C (pH 6). We then identified mutant residues in the anti-IL-8 mAb framework and CDRs that would significantly disrupt hydrophobic, positive, and negative patches (Supplementary file 1), potentially influencing protein-protein interactions and self-association.

### 2.3 Patch analysis of anti-IL-8 mAb mutants

**Fig. 2:**
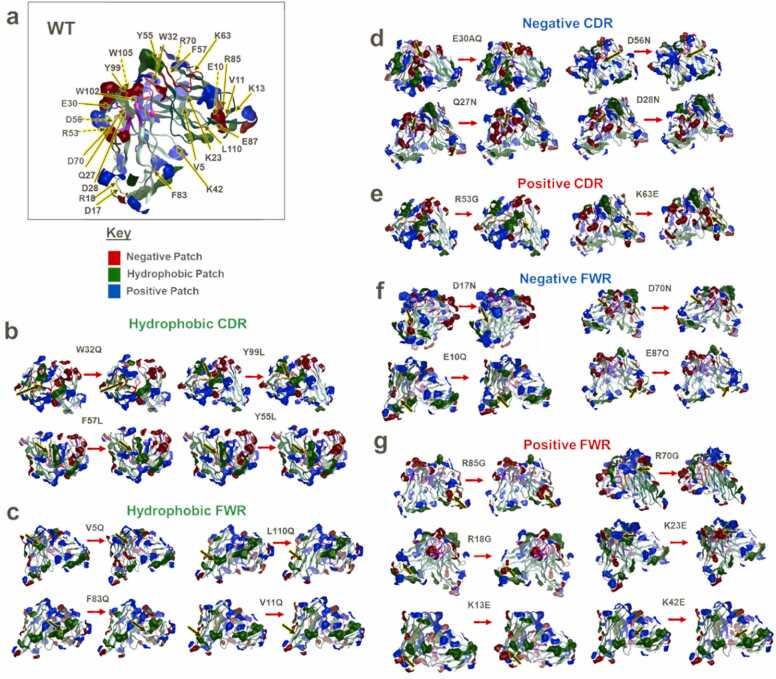
**Patch analysis of WT (a) and mutant Fv homology models disrupting hydrophobic patches (green- c-b), negative patches (red- d, f), and positive patches (blue- e, g).** VL (light green), VH (dark green), heavy chain CDRs (red), and light chain CDRs (purple) are shown. The WT (left) and corresponding mutant (right) are represented for each molecule. Arrows show the location of the single point mutation. Dashed lines represent residues behind the field of view.

We explored the effects of single point mutations on the anti-IL-8 mAb charge and hydrophobic patch distributions, by introducing Fv point mutations. Employing strategies targeting positive, hydrophobic, and negative patches, we observed changes in electrostatic surface potentials following framework region and CDR mutations (Fig. 2). [18], [19], [20] The anti-IL-8 mAb Fv carries a net positive charge (+0.05 C, pH 6.0), with heterogeneous surface charge distribution, resulting in asymmetry between heavy and light chain net charges (3.93 C and −1.23 C, respectively). Patch analysis of the WT Fv revealed significant hydrophobicity (520 Å^2^) with prevalent surface coverage by positive patches (blue).

Residues with the highest contributions to positive (blue), negative (red), and hydrophobic (green) patches were identified from patch analysis of the anti-IL-8 mAb WT Fv homology model. Key residues for sequence-based modification included those contributing to positive (blue) (e.g.*,* K42, K23, R18, K13, R85 and R70 for the framework region, and R53 and K63 for CDRs), negative (red) (e.g.*,* D70, E10, E87, D17 for the framework region, and E30A, D56, Q27 and D28 for CDRs) and hydrophobic (green) (e.g.*,* F83, L110, V11, V5 for the framework region, and W32, Y99, F57 and Y55 for CDRs, Supplementary file 1) patches.

Global patch analysis of anti-IL-8 mAb Fv mutants (Fig. 2) revealed that R→G and K→E mutants (positive patch-targeting [18]) exhibited reduced positive patch coverage, while V/W/L/F→Q and F/Y→L mutants (hydrophobic patch-targeting [19]) showed reduced hydrophobic patch area coverage. D→N and E→Q mutants (negative patch-targeting [20]) displayed a reduction in negative patch area coverage. However, these mutations did not exclusively impact the targeted patch, with depletion and enhancement of neighbouring patches being observed.

Next, we computed physicochemical molecular descriptors for all candidate mutant Fv homology structures, some of which have shown prior positive or negative correlations with viscosity (Supplementary file 2). [22], [23] We found that charge-based mutant Fvs resulted in changes in predicted net charge, ensemble charge (*ens_charge*), zeta potential, isoelectric point (*pI_seq* and *pI_3D*), and light and heavy chain charge imbalance (*Fv_chml*). Significant differences in hydrophobicity descriptors were observed with mutants targeting hydrophobic patches (Supplementary file 2). [19] The therapeutic antibody profiler (TAP) [24], [25] was used to predict developability risk for each candidate mutant (Supplementary file 3). All mutants were amber-flagged for hydrophobic patches near CDRs, red-flagged for a positive patch targeting mutant (K42E) and a hydrophobic patch targeting mutant (W102Q). We evaluated charge symmetry, with three positive patch-targeting mutants (K42E, R18G and R53G) being amber flagged. From TAP analyses, we identified specific mutants (W102Q, R18G, R53G and K42E) as the ‘*least developable*’ candidates.

#### 2.3.1 Light chain-heavy chain charge separation

We observed shifts in charge distribution profiles reflected in *Fv_chml* and *FvCSP* descriptors, which indicate charge imbalances between heavy (VH net charge) and light chains (VL net charge). In all cases, VL net charge was negative (−1.23 C for the anti-IL-8 mAb WT) and VH net charge was positive (+3.93 C for the anti-IL-8 mAb WT). Since *FvCSP* is a product of VH and VL charges, we noted a larger difference with negatively-charged VL mutants. For example, with VL and VH charges at − 1 C and − 4 C, respectively, a 1 C drop in VL net charge reduced *FvCSP* from − 4 to − 8 C. A 1 C reduction in VH net charge reduced *FvCSP* from − 4 C to − 3 C. When net charges of either VL or VH chain were 0, *FvCSP* was 0, potentially misinterpreted as no existing charge differences between chains. [26] This underscores the importance of *Fv_chml* descriptors, which subtract VL charge from VH charge.

Mutants targeting negative patches in VL, [20] resulted in a ≤ 0.91 C charge increase, with a similar increase seen for VH mutants. For nearly all VL D*→*N mutants, we observed increased *FvCSP* and reduced *Fv_chml*, suggesting enhanced charge symmetry between VH and VL chains. However, VH E*→*Q mutants showed a reduction in *FvCSP* and increased *Fv-chml*, indicating increased charge imbalance, absent in Q27N.

Conversely, mutants targeting positive patches [18] exhibited increased VL negative charge (K42E: −1.9 C), resulting in more negative *FvCSP* and increased *Fv_chml* descriptors. VH mutants had reduced VH charge, approaching VL charge (∼3 C), leading to increased *FvCSP* and decreased *Fv_chml*, reflecting reduced charge imbalance between VL and VH. Mutants primarily targeting hydrophobic patches [19] resulted in *FvCSP* and *Fv_chml* comparable to anti-IL-8 mAb WT. These data emphasise that single-point mutations in VL versus VH depend on parent WT mAb initial charge symmetry and must be evaluated on a case-by-case basis.

#### 2.3.2 Triage of candidate mutants

The anti-IL-8 mAb mutant panel was ranked using a summed normalised score, guiding our selection of mutants for expression and physicochemical measurements (Supplementary file 5). We selected two hydrophobic-targeting mutants, four negative patch-targeting mutants, and two positive patch-targeting mutants for expression and subsequent formulation at high concentration (>200 mg/mL). We anticipated that the W32Q mutant, disrupting hydrophobic patches, would significantly reduce viscosity relative to anti-IL-8 mAb WT, while mutants disrupting positive patches (R53G and K42E) would likely show increased viscosity at high concentrations.

### 2.4 Biophysical parameters of the expressed mutant panel

We aimed to establish a comprehensive measurement pipeline for the expressed anti-IL-8 mAb mutant panel, correlating these observations with predicted physicochemical descriptors and viscosity-related parameters to understand factors underlying elevated viscosity in high-concentration antibody formulations. We confirmed the sequence identity and post-translational modifications of WT and mutant anti-IL-8 mAb via mass spectrometry-based peptide mapping (Supplementary file 6). Additionally, all mutants met the monomeric purity threshold by aSEC (analytical size-exclusion chromatography≥ 95 %,) (Supplementary file 6). Apart from W32Q (CDRH2 mutant), mutants retained antigen binding affinity and kinetics equivalent to WT anti-IL-8 mAb (Supplementary file 6). Next, we analysed the mutants for their hydrophobic, colloidal, electrostatic, and conformational properties.

#### 2.4.1 Electrostatic properties of the anti-IL-8 mAb mutant panel and the correlation between predicted and experimental parameters

Therapeutic antibodies are typically formulated at high concentrations in the pH 5.2–6.3 range, where the constant regions exhibit a positive net charge, driving repulsive interactions. Variations in charges within the variable region can influence viscosity at high concentrations. [23], [27]

**Fig. 3:**
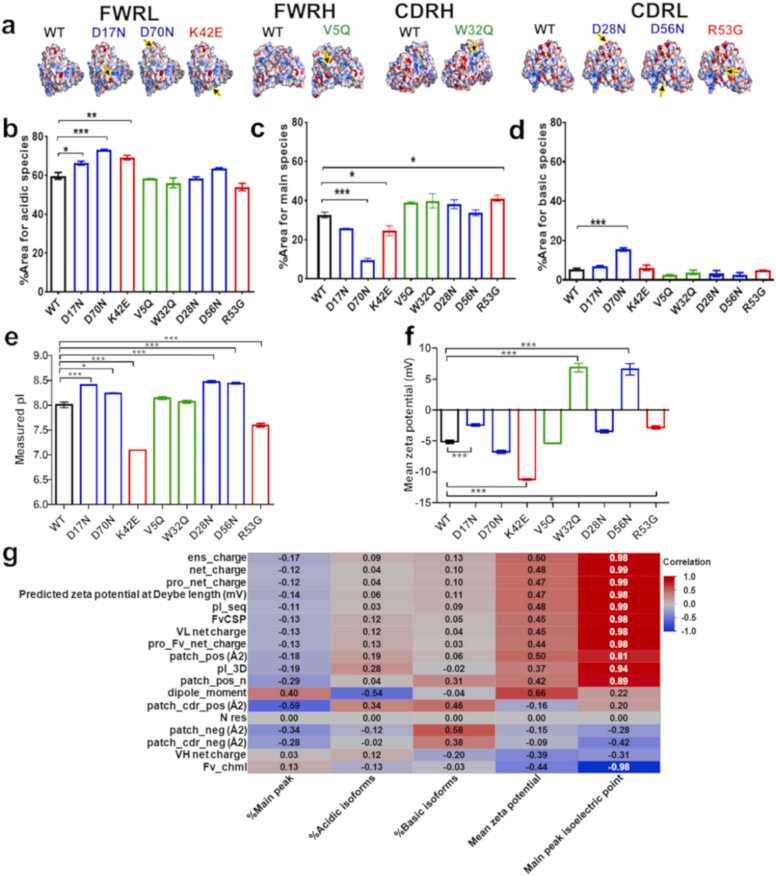
**Negative and positive patch disrupting mutants show a strong correlation between predicted and measured PI**. **a,** Poisson-Boltzmann surfaces were mapped onto all anti-IL-8 mAb mutant Fv models, categorised by location, demonstrating the impact of single-point mutations on electrostatic distributions around the mutation site (marked by an arrow). **b-f,** Charge-based profiling of anti-IL-8 mAb mutant panel with cIEF (N = 2) **g,** correlation analyses of zeta-potential showed a weak corelation between the in silico descriptor and experimental zeta-potential (N = 3) (R=0.47). Strong positive correlations were observed for pI_seq and pI_3D (sequence and structure based isoelectric point predictions) with the experimental isoelectric points (R=0.99 and 0.94, respectively). A one-way ANOVA with Dunnett’s comparison test was used to compare anti-IL-8 mAb mutants with the WT. * ** denotes a P < 0.001, * * P < 0.01. Non-significant differences are not represented. R values were computed from simple linear regression of in silico molecular descriptors and experimental values.

Two strategies were employed to generate mutants, targeting positive and negative patches. Therefore, we evaluated electrostatic properties of the mutant anti-IL-8 mAb panel and correlated them with viscosity-concentration profiles. Predicted net charge, isoelectric point (pI), and zeta-potential based on anti-IL-8 mAb Fv (Supplementary file 2) were compared with experimental measurements (Fig. 3).

Spatial charge distributions of mutants were visualised with two-dimensional maps (Supplementary file 4) to track changes resulting from single point mutations. For example, the D17N mutation led to the loss of a 30 Å^2^ negative patch and a similarly sized hydrophobic patch, with adjacent positive patch surface distributions shifting (WT 2D map numbers 9 and 18 *→* D17N 2D map numbers 10 and 6). Changes in measured isoelectric point (pI) were observed, with increased charge for negative patch disrupting mutants, decreased charge for positive patch disrupting mutants, and no significant changes for hydrophobic patch disrupting mutants. (Fig. 3**e**). The majority of anti-IL-8 mAb molecules displayed a negative zeta potential (Fig. 3f), except for W32Q and D56N, which had a positive zeta potential. D17N and R53G showed significant increases in zeta potential, while K42E (a positive patch-disrupting mutant) exhibited a reduced zeta potential relative to the WT.

We correlated experimental charge data with predicted *in silico* zeta potential and pI descriptors using linear regression(Fig. 3g). While no correlation was found between the predicted and experimental zeta potential (Pearson correlation coefficient, R =0.47), a strong positive correlation was observed for sequence- and structure-based pI descriptors and measured pI (R=0.99 and 0.94, respectively).

#### 2.4.2 Hydrophobicity of the mutant anti-IL-8 mAb panel and the correlation between predicted and measured parameters

As stated above, protein-protein interactions and self-association at high formulation concentrations, potentially lead to elevated viscosity. Here, we explored the role of alterations in hydrophobic surface area coverage as a strategy to reduce viscosity, correlating predicted hydrophobicity descriptors with experimental measures. [19]

**Fig. 4:**
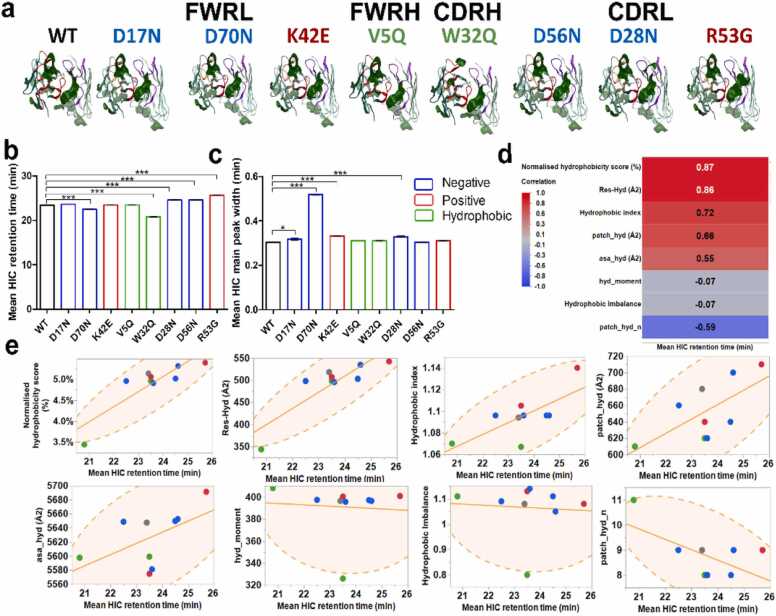
Hydrophobic Interaction Chromatography (HIC) of the WT and mutant anti-IL-8 mAb panel and correlation with predicted hydrophobicity molecular descriptors. a, protein patch surface maps are depicted for all anti-IL-8 mAb mutants, filtered for hydrophobic patches (green). b, HIC retention time and c, corresponding HIC peak widths for the anti-IL-8 mAb mutants (N = 2). Statistical significance was assessed with a one-way ANOVA with Dunnett’s comparison test to WT (*** denotes a P < 0.001, * P < 0.1). Non-significant differences are not represented. d, correlation analysis between in silico hydrophobicity descriptors and experimental retention time for anti-IL-8 mAb mutants. Strong correlations (R ≥±0.8) are labelled in white. e, scatterplots showing linear correlations for anti-IL-8 mAb mutants with P = 0.95 bivariate density ellipses. All antibodies are colour-coded according to mutants targeting positive (red), negative (blue), and hydrophobic (green) patches.

Using Hydrophobic Interaction Chromatography (HIC), we probed changes in hydrophobicity among the anti-IL-8 mAb mutant panel. We anticipated reduced hydrophobicity for mutants targeting hydrophobic patches, and smaller changes for those targeting charged patches (Fig. 4 and Supplementary file 4). Indeed, we observed a shorter retention time for W32Q, consistent with predicted reduction in hydrophobicity. Unexpectedly, D70N also showed reduced retention time compared to WT, contrary to predictions. Interestingly, V5Q, predicted to have reduced hydrophobicity, exhibited longer retention time. However, this contradicted predictions, possibly due to differences in targeted hydrophobic patch sizes. Mutants in the CDRL region (D28N, D56N and R53G) showed longer retention times, correlating with spatial hydrophobicity profiles (Supplementary file 2 and 6). Using correlation analysis, we found a strong potential correlation (R=0.87) between normalised hydrophobicity score and summed residue contributions to hydrophobic patch area (*res _hyd*), offering insights into ranking the hydrophobicity of anti-IL-8 mAb mutants.

#### 2.4.3 Conformational stability of the mutant anti-IL-8 mAb panel

We employed intrinsic fluorescence DSF to measure the effects of single-point mutations on anti-IL-8 mAb conformational stability. We used first derivative 350/330 nm ratio traces and scattering traces were used to calculate the unfolding onset temperature (T_onset_), melting temperatures, and the temperature of aggregation onset (T_agg_). Overall, mutants showed comparable thermal stability, except for W32Q and R53G (Supplementary file 6). W32Q (hydrophobic patch-targeting) exhibited decreased T_onset_, T_agg_ and T_m1,_ suggesting reduced thermal stability. This reduction may stem from the disruption of a large hydrophobic patch (150 Å^2^), critical for stabilising the CDRH2 domain secondary/tertiary structure. R53G (positive patch-disrupting mutant), also showed reduced thermal stability (decreased T_onset_).

#### 2.4.4 Propensity for interactions promoting self-association

**Fig. 5:**
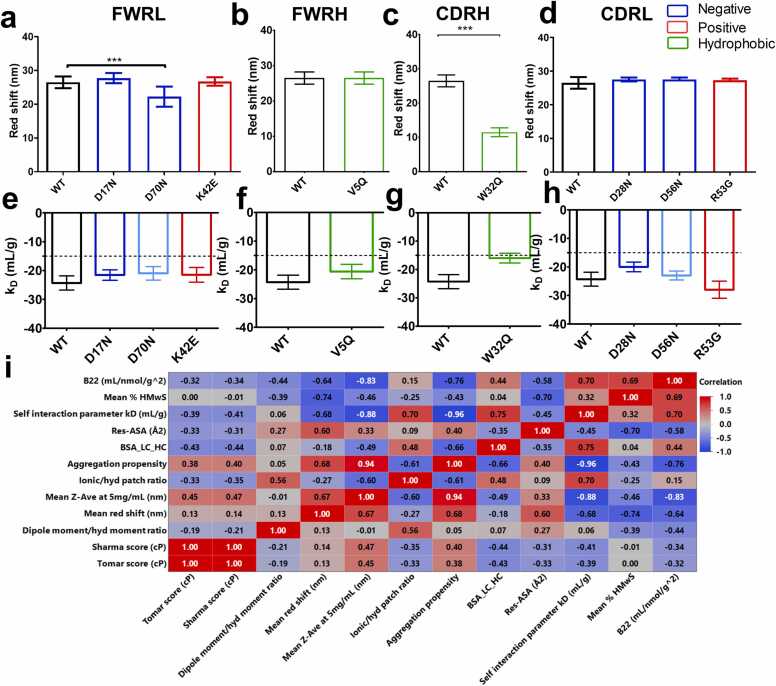
WT and mutant anti-IL-8 mAb panel propensity for self-association as measured with AC-SINS and self-interaction parameter (kD), categorised by mutation location, and coloured by mutation strategy. All antibodies are colour-coded according to mutants targeting positive (red), negative (blue), and hydrophobic (green) patches. a-d, corresponding AC-SINS data (N = 4) e-h, the self-interaction parameter calculated from analysis of diffusion coefficients (N = 3) measured by DLS (1–30 mg/mL). A dotted line at − 15 mL/g represents an arbitrary threshold for k_D_. A one-way ANOVA with Dunnett’s comparison test to WT Non-significant differences are not represented. No significant differences were identified for k_D_ values between mutants and WT, but W32Q showed a less negative mean k_D_. i, colloidal interaction experimental results (k_D_ and mean red shift) and hydrodynamic size (Z_ave) and the % high molecular weight species (soluble aggregates) were cross-correlated with in silico molecular descriptors describing the structural accessibility (Res-ASA and BSA_LC_HC), the charge/hydrophobicity ratios, and the aggregation propensity scores. These were selected describe the intrinsic biophysical profile of the anti-IL-8 mAb mutants and their self-interaction propensity. Strong correlations (R ≥ ± 0.8) are labelled in white.

AC-SINS and high throughput diffusion self-interaction parameters (*k*_*D*_) were used to determine diffusion coefficients (Supplementary file 6) as surrogate measures of propensity for protein-protein interactions (Fig. 5).

AC-SINS detects self-association by red shifts in UV-Vis spectra (Fig. 5**a**), indicating increased particle size. Compared to the anti-IL-8 mAb WT, D70N and W32Q mutants showed reduced red shift in absorbance measurements (Fig. 5**b, d**), suggesting decreased self-association propensity.

The k_D_ parameter, indicative of protein-protein interaction risk, was comparable to WT for all mutants except W32Q, which, although not statistically significant, had a notably less negative k_D_, signifying reduced short-range attractive self-interactions. [28] (Fig. 5 h). This was consistent with a less negative second virial coefficient (B22) for W32Q (Supplementary file 6). Overall, both AC-SINS and k_D_ data suggest a reduced aggregation risk for W32Q.

TANGO aggregation propensity scores, serving as *in* silico predictors of aggregation, negatively correlated with k_D_, soluble aggregates (-high molecular weight species, %HMwS) and hydrodynamic diameter (Z-Ave) (Fig. 5**j**), indicating solvent exposure plays a key role in driving mAb self-association. [29]

#### 2.4.5 Viscosity-concentration profiles of anti-IL-8 mAb mutants

**Fig. 6:**
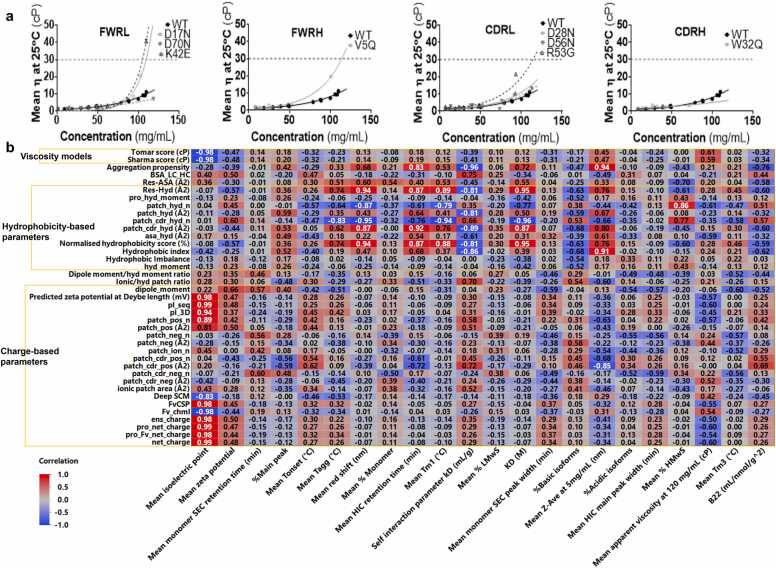
**anti-IL-8 mAb mutant panel viscosity correlation heatmap. a,** Mean apparent viscosity-concentration profiles measured at 25 °C for all anti-IL-8 mAb variants (<120 mg/mL). Dotted grey line at 30 cP represents ‘acceptable viscosity’. All measurement data were fitted to exponential growth equations through a least squares fitting method. **b,** correlation heat map values are reported with strong correlations (R > ± 0.8) in white font.

We evaluated the viscosity of the anti-IL-8 mAb panel at various concentrations using microfluidic rheometry and compared their viscosity profiles to the WT molecule. Non-Newtonian behaviours were not observed across shear sweep experiments (data not shown), so average apparent viscosities were determined with exponential growth fits **(**Fig. 6**a)**. Among the mutants, D70N (negative patch-disrupting FWRL) and W32Q (hydrophobic patch-disrupting CDRH), showed reduced viscosity compared to WT.

#### 2.4.6 Correlating in silico descriptors with biophysical characterisations

We correlated all molecular descriptors used for designing anti-IL-8 mAb mutants with their biophysical characteristics (Fig. 6**b**). For charge-based *in silico* descriptors, the strongest correlations were observed with mean *experimental pI* (Fig. 3). Weak negative correlations were noted between *net charge* and *pI_seq* and the *mean apparent viscosity* (R= −0.6). A strong negative correlation was found between *patch_cdr_pos area* and the *mean hydrodynamic diameter* (R= −0.85).

Regarding hydrophobicity-based descriptors, strong correlations were observed with *HIC retention time* (Fig. 4), affinity (KD), AC-SINS red shift and the self-association parameter k_D_. Some strong correlations were also noted between *res _hyd* (R=0.89), *normalised hydrophobicity scores* (R=0.88), and *hydrophobic patch counts* (Fv and near CDRs) (R=−0.94 and −0.79, respectively) with the *Tm*_*1*_ *unfolding temperatures*, suggesting the influence of exposed hydrophobic patches on conformational stability of these anti-IL-8 mAb mutants. The number of hydrophobic patches near CDRs was correlated with the *temperature of aggregation onset (T*_*agg*_*)*. Additionally, a correlation was observed between the *number of hydrophobic patches* and *the % high molecular weight species* from the aSEC analysis (R=0.86), aligning with hypotheses on the impact of hydrophobic interactions in the mechanism for aggregation. [30], [31] Strong correlations were observed with the TANGO aggregation propensity scores to hydrodynamic diameter (R=0.94), HIC retention time (R=0.83) and k_D_ (R=−0.8). Finally, strong negative correlations were seen with Tomar and Sharma viscosity models, and experimental pIs (−0.98), which is expected as these models are primarily based on charge-related parameters.

## 3 Discussion

In this study using a combination of computational and experimental approaches we assess how single-point mutations affect surface exposed electrostatic parameters, hydrophobicity, colloidal, and viscosity behaviour at high formulation concentration in an anti-IL-8 model antibody. We applied three sequence-structure based strategies to design mutants based on targeting charged (positive and negative) and hydrophobic patches, so that we could compare their effectiveness in predicting developability issues. [32], [33] We were particularly interested in controlling protein-protein interactions known to govern elevated solution viscosity at high formulation concentrations.

Our *in silico* predictions of anti-IL-8 mAb physicochemical descriptors revealed notable changes in surface-exposed charged and hydrophobic patches. Mutations in the CDR have previously been shown to reduce mAb viscosity and antigen affinity loss, so we expanded our screening to include mutants in the anti-IL-8 mAb heavy and light chain framework regions (Supplementary file 6) [10], [34] Except for W32Q (a CDRH mutation), seven mutants (87.5 %) maintained binding affinities for IL-8 equivalent to the WT anti-IL-8 mAb (3.9 nM). W32Q, however, exhibited a five-fold reduction in hydrophobic patch area coverage, suggesting a critical role for tryptophan in antigen binding. This observation aligns with prior studies, where substituting the tryptophan with non-polar and polar amino acids retained binding affinity for phenylalanine mutants, emphasising the importance of the aromatic ring in antigen binding. [4]

The monomeric purity and aggregation status were acceptable for all anti-IL-8 mAb mutants and equivalent to the WT. Overall, point mutations in the anti-IL-8 mAb positive and negative patches significantly altered surface potential, inferred colloidal stability, charge heterogeneity and net charge (Fig. 3).

### 3.1 Charge-disrupting mutants do not mitigate for elevated viscosity at high-concentration

Adjusting the electrostatic surface potential of mAbs is routinely explored during formulation development, focusing on buffer composition, which alters the excluded volume of the protein in solution (the electroviscous effect). [35], [36] Chow *et al.* [18] demonstrated viscosity reductions in an IgG4 Fab fragment by reducing charge imbalance across the Fv (R→G and K→E mutants), indicating the impact of positive patch disruption on protein-protein interactions. Conversely, Apgar *et al.* [20] observed viscosity reduction in mAbs by reducing negative charge, as evidenced by viscosity reduction for D→E to N→Q mutants. [32]

In this study, the anti-IL-8 mAb WT Fv homology construct exhibited a high proportion of positive patches, indicating a potentially high baseline electrostatic potential with developability risks. We used various *in silico* molecular descriptors (*supplementary information*) to assess developability risks arising from anti-IL-8 mAb electrostatic properties. We found negative patch-disrupting mutants reduced charge imbalance [23], increased net charge, [37] and ensemble charges, [26] which have previously been correlated with viscosity reduction. These mutants also exhibited higher pIs, potentially enhancing anti-IL-8 mAb colloidal stability. Conversely, positive patch-disrupting mutants showed reduced ensemble charges and significantly decreased pIs, suggesting diminished colloidal stability.

Contrary to the predicted net charges and surfaces charges, Zeta potential valuesfor most anti-IL-8 mAb mutants (except W32Q and D56N which had positive zeta potentials) revealed predominantly negative zeta potential values at pH 6.0, consistent with a net negative surface charge observed in the WT anti-IL-8 mAb The discrepancies between computed predicted charges (+22.68 C for WT Fv at pH 6) and the negative measured zeta potentials can be attributed to multiple factors. One is a lack of accurate modelling of buffer components, affecting surface bound ions. Another is not accounting for other potential species in the system, such as aggregates or fragments carrying different surface charges. Furthermore, charge computations did not account for multiple molecules in the system and thereby neglected electrostatic effects from protein-protein interactions. These factors may also explain the lack of correlation to isoelectric points, which were measured at a much lower concentration (0.4 mg/mL versus 5 mg/mL for cIEF and zeta potential, respectively). The positive patch disrupting mutant, R53G, had more positive zeta potential but the second-lowest pI value in the mutant panel. Potential clustering of this mutant even at 5 mg/mL could be increasing the surface charge in this instance. Conversely, the K42E mutant exhibited a significantly lower zeta potential compared to the WT, supporting the notion that mutants disrupting positive patches tend to have more negative zeta potentials.

Therapeutic antibody profiler (TAP) predictions provide charge-based metrics for the anti-IL-8 mAb mutants, with flags indicating charge symmetry primarily in R53G and K42E positive patch targeting mutants (Supplementary file 1). However, all TAP scores for both positive and negative disrupting mutants fell within an ‘acceptable’ range, suggesting limited discriminatory power of TAP. This lack of differentiation in TAP scores has been noted in previous studies, highlighting potential limitations in its applicability for comprehensive mAb characterization. [8]

### 3.2 Mutants targeting hydrophobic patches exhibit altered viscosity

Research strategies have explored strategies beyond neutralising charged patches to reduce hydrophobic interactions, for mitigating high concentration mAb stability and viscosity risks. [19] We computed hydrophobicity-based descriptors for correlation with viscosity and developability, and compared these with HIC retention times (Fig. 4). Our analyses revealed a reduced hydrophobicity for W32Q, consistent with its predicted decrease in solvent-accessible hydrophobic patch area. However, smaller changes in hydrophobic patch area coverage were undetectable via HIC. Mutants with the lowest HIC retention times demonstrated lower solution viscosities (Fig. 6), indicating a significant role for hydrophobic interactions in driving self-association. Strong correlations were observed between hydrophobic-based *in silico* descriptors and the observed HIC retention times for the anti-IL-8 mAb mutant panel, highlighting the predictive power of these descriptors in understanding viscosity behaviour.

Various research efforts have explored colloidal self-interaction as part of early mAb developability assessments. [38] The B22 or A2 second virial coefficient and the self-interaction parameter, k_D_, are key metrics capturing the thermodynamic effects of self-associating mAbs at dilute mAb concentrations. [39] Negative values for B22 and k_D_ indicate attractive protein-protein interactions, associated with decreased formulation stability and increased solution viscosity at high concentrations. [14], [18], [40] In this study, all anti-IL-8 mAb mutants exhibited negative k_D_ values, with the W32Q mutant showing a less negative k_D_, aligning with its reduced hydrophobicity. The AC-SINS assay further supported reduced self-association propensity for W32Q, consistent with the measured k_D_ (Fig. 5). Trends were observed between colloidal parameters measured at lower anti-IL-8 mAb concentrations and viscosity-concentration profiles (<120 mg/mL), indicating reduced self-association propensities and viscosities for D70N and W32Q.

Most mutants showed similar unfolding temperatures to the WT, except for W32Q, suggesting a critical role for tryptophan in maintaining a large hydrophobic patch in the CDRH2, which impart stability which is lost upon mutation (Supplementary file 6). This reduced thermal stability also aligns with the observed reduction in antigen binding for W32Q. .

**Fig. 7:**
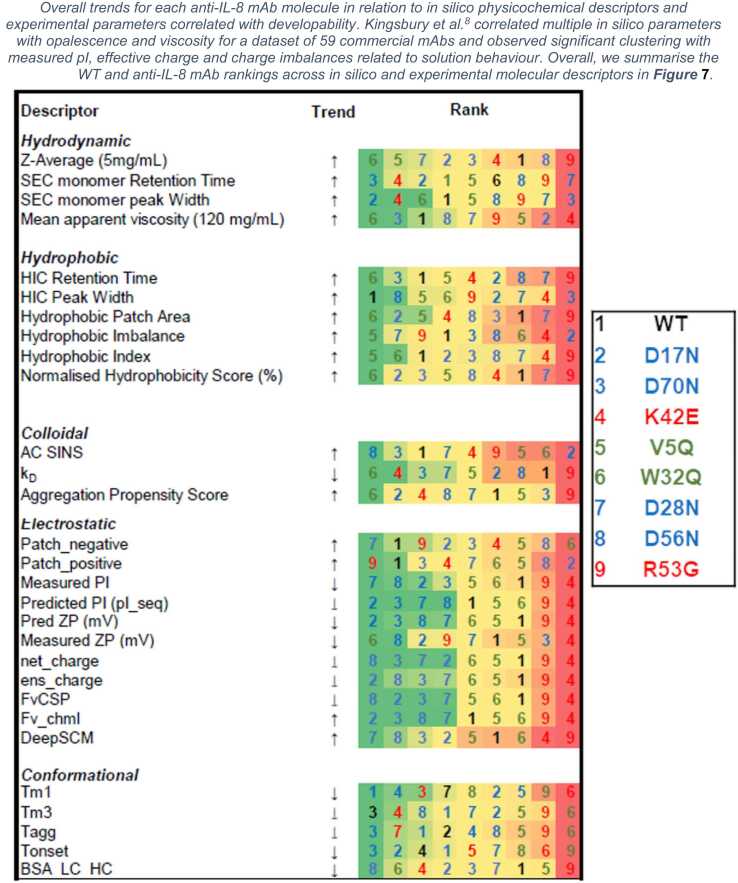
**Ranking matrix for the anti-IL-8 mAb mutant panel** A colour-coded from min-max ranking in order of decreasing developability and categorised by experimental parameters and molecular descriptors.

This is the first study that enables comparison of predictive and empirical approaches to understand the role of electrostatic and hydrophobic patch targeting in altering viscosity in the same mAb molecule. While our findings offer valuable insight into these strategies, there are associated limitations. Unlike previous reports, [15] we did not observe specific trends in viscosity reduction based on mutation site (CDR versus FWR) in the anti-IL-8 mAb scaffold. Given the variability in charge and non-polar patch distribution among individual mAbs, generalised approaches to reduce molecular interactions driving self-associations may not be suitable and require a systematic design-build-test-learn approach. While we explored single-point mutations, sequence engineering may require multiple mutation sites for improved developability. Previous studies have shown enhanced viscosity reduction through combined substitutions in both VH and VL regions. [41] Our computational simulations focused on Fv models and did not consider the influence of hinge and constant domains on biophysical characteristics such as charge and hydrophobicity. Also, additional investigations are required to ascertain the impacts of non-CDR mutations on downstream biological properties of mutant mAbs (e.g., immunogenicity, half-life).

Early-stage assessment of pharmaceutical candidates is crucial for guiding decisions on clinical translation. Various industry-wide criteria are used to triage lead biomolecules, and the use of data-driven sequence-engineering strategies to optimise lead candidates represents a growing field. Our investigation shows that trends observed from molecular descriptors to biophysical properties have a strong dependence on the mutation strategy employed. We find that mutations with significant reductions in hydrophobic patches significantly improved mAb solution viscosity, suggesting the predictive power of hydrophobic-based descriptors. However, mutations altering electrostatic patch coverage alone were insufficient to impact viscosity, irrespective of mutation site. Integrating deep learning approaches holds promise for deeper mechanistic insights into mAb developability, yet challenges such as wider data availability in the pre-competitive research landscape remain. Our study highlights the importance of considering both sequence-based and structural alterations in optimising mAb developability characteristics.

## 4 Materials and methods

### 4.1 Computational methods

*In silico* homology modelling and antibody molecular descriptor calculations were performed in the Molecular Operating Environment (MOE) software, version 2020.0901 (Chemical Computing Group, Montreal, Canada).

#### 4.1.1 Homology modelling

Full sequences of the heavy and light chains of an immunoglobulin G1 (IgG1) wild-type (WT) molecule were inputted as FASTA format into the *MOE sequence editor* and annotated with a Kabat numbering scheme. The *Antibody modeller* in MOE (version 2020.0901) was used to search for similar sequences with solved antibody structures as a template for homology constructs. The variable fragment (Fv) of anti-IL-8 mAb is published as PDB ID: 5OB5 (fAb complex with GroBeta). Fv fragments and full IgG structures were modelled by selecting ‘variable domain’ and ‘immunoglobulin’ model types, respectively. The immunoglobulin model type uses the 1IGY PDB structure as a template to model the Fc region. A refinement gradient limit value of 1 was applied, and C-termini were capped with neutral residues, and superimposed to confirm alignment of structures. Partial charges were added to all atoms, and energy minimization performed using the AMBER10:EHT default forcefield. The Protein Silo (PSILO) database was used to locate sites of hydrogen bonding and other potential interactions with the GroBeta ligand in complex with the Fv.

#### 4.1.2 Patch analysis and identification of the mutant panel

The protein patch tool in MOE was applied to the WT Fv homology construct to identify electrostatic and hydrophobic surface patches. To aid visualisation of smaller surface patches, we set the following parameter thresholds: hydrophobic cut-off: ≥ 0.09 kcal/mol, hydrophobic min area: ≥ 30 Å^2^, charge cut-off: ≥ 30 kcal/mol/C, charge min area: ≥ 30 Å^2^, probe sphere radius: 1.8 Å. The residue contribution to the surface patches was analyzed using the *Protein Properties* tool, selecting the ‘*res _hyd*’, ‘*res _pos*’ and ‘*res _neg*’ descriptors. The top scoring residues were then selected as candidate residues for mutations, excluding terminal residues (Supplementary file 1). Three approaches were implemented to alter solvent-accessible charged patches, by **i)** substituting aromatic hydrophobic residues to leucine or glutamine (L or Q), [19] and **ii)** substituting positively-charged residues (e.g., N or R) to glutamic acid or glycine (E or G), [18] and **iii)** substituting negatively-charged glutamic acid or aspartic acid (E or D) to positive residues (e.g., N). [20] We used *Residue Scan* in MOE to introduce point mutations in the WT anti-IL-8 mAb Fv IgG1 sequence.

**Predicted physicochemical descriptors.** We computed a range of physicochemical descriptors (Supplementary file 2) for each Fv model using the MOE Protein Properties tool. A NaCl concentration of 0.1 M was used to mimic the ionic strength of the formulation buffer (pH 6). Hydrophobic imbalance and buried surface area, Fv_chml values were generated through BioMOE (version 2021–11-18, Chemical Computing Group, Montreal, Canada) for models protonated to pH 6 using the QuickPrep tool.

**TANGO aggregation propensity** (http://tango.crg.es/tango.jsp). [42], [43] TANGO aggregation was used to predict the sequence-based propensity for beta-sheet formation for all mutants.

**Ranking anti-IL-8 mAb mutants.** Candidate anti-IL-8 mAb mutant variants were ranked using a min-max normalisation method to triage mutants for further investigation. Physicochemical descriptors were selected based on prior correlations with viscosity and weighted evenly. Hydrophobic index, TANGO aggregation propensity, the normalised hydrophobic score (proportion of exposed hydrophobic areas (Res_hyd) to the total exposed surface area (Res_ASA)), zeta potential, buried surface area between heavy and light chains (BSA) and the ensemble charge (ens_charge) were parameters used for ranking. Descriptor values were normalised between 0–1 (Eq. 3).

(3) $$\mathrm{NDV}=\;\frac{x-x_{\min}}{\left(x_{\max}-x_{\min}\right)}$$

Where *NDV* is the normalised value for a mutant, *x* is the actual descriptor value for a mutant, and *x*_*min*_ and *x*_*max*_ are the minimum and maximum values found in the mutant panel for that descriptor.

A normalised score was calculated by summing each normalised descriptor value (**Equation 4 A**), or summing 1- normalised descriptor value for descriptors correlating negatively with elevated viscosity (Eq. 4B). Therefore, a lower normalised score overall represented a reduced hypothetical viscosity.

(4A) $$\mathrm{Normalised score}=\mathrm{SUM}(\mathrm{ND}V_{\mathrm{HI}}+\;{\mathrm{ND}V_{\mathrm{TANGO}}+\mathrm{NDV}}_{\mathrm{Tomar}}+\mathrm{ND}V_{\mathrm{Sharma}}+\mathrm{ND}V_{\mathrm{Normalised hydrophobic score})\;}$$

(4B) $$\mathrm{Normalised score}=\mathrm{SUM}((1-\mathrm{ND}V_{\mathrm{zeta}})+(1-{\mathrm{NDV}}_{\mathrm{BSA}})+(1-\mathrm{ND}V_{\mathrm{en}s\_charge}))$$

***DeepSCM*** (https://github.com/Lailabcode/DeepSCM). [32] We used the spatial charge map to rank mutants by calculating the charge of side chain atoms of exposed residues of a homology Fv model over molecular dynamics simulations. [32], [44] We inputted anti-IL-8 mAb IgG Fv sequences as separate heavy and light chain FASTA files and the code was ran in a terminal.

### 4.2 **Protein Expression and Purification**

Chinese Hamster Ovary (CHO) K1 GS-KO (glutamine-synthetase-knockout) cells were used for expression of the anti-IL-8 mAb panel. Sequences for anti-IL-8 mAb variants underwent codon optimisation and plasmid generation by Atum Biosciences (Newark, California, USA). The heavy and light chain genes were inserted into Leap-in Transposase® pD2500 vectors with the CMV promoter including glutamine synthetase (for selection) and heavy and light chain insertions were nucleofected into CHO cells. Cells were maintained under selection conditions as stable pooled cultures. A fed-batch production process was employed over 15 days, with glucose and supplementary amino acid feeds added at various intervals. Expression media were harvested and the supernatant clarified by centrifugation at 4 °C (4000 g for 20 min) and sterile-filtered. Protein L chromatography on an ÄKTA Avant 150 system (Cytiva, Danaher, USA) was used for purification, followed by a cation exchange polishing step to achieve ≥ 95 % monomeric purity. The purified mAbs were concentrated, diafiltered and buffer exchanged into formulation buffer containing histidine, trehalose, and arginine (pH 6) to a final concentration of ≥ 100 mg/mL using the Ambr Crossflow system (Sartorius, Germany). All mutants showed full solubility at 25 °C with no liquid-liquid phase separation observations.

### 4.3 Analysis of the WT and mutant anti-IL-8 mAb panel biophysical parameters

#### 4.3.1 Analysis of anti-IL-8 mAb identity and purity

Peptide mapping was used to confirm the full sequence identity for the anti-IL-8 mAb WT and mutant panel. The monomeric purity of WT and mutant anti-IL-8 mAb variants was analysed by analytical size exclusion chromatography (aSEC) with UV detection (*supplementary information*).

#### 4.3.2 Hydrophobic interaction chromatography

Hydrophobicity of the anti-IL-8 mAb panel was assessed via hydrophobic interaction chromatography (HIC) with UV detection. A PolyLC PolyPROPUL 4.6×100 mm column was used on an Agilent 1260 series HPLC (Agilent, California, US). The mobile phase A contained high salt (1.3 M ammonium sulfate) in a potassium phosphate buffer (50 mM, pH 7), with stepwise gradient segments. All samples were analysed at a concentration of 1 mg/mL (with a 5 μL injection) at a flow rate of 0.7 mL/min and detected at 214 and 280 nm wavelengths.

#### 4.3.3 Electrophoretic light scattering

A Malvern Zetasizer (Malvern Panalytical, Malvern, UK) with a 633 nm laser was used to measure zeta potential of all samples at 5 mg/mL by electrophoretic light scattering. The default settings included an equilibration time of 120 s, automatic attenuation and 10–100 measurement runs. A 60-second pause was added between measurements and three technical replicate measurements were performed. Both the WT and mutant anti-IL-8 mAb molecules were prepared in formulation buffer and filtered prior to analysis.

#### 4.3.4 Diffusion self-interaction parameter

We used a stunner (Unchained Labs, CA, USA) dynamic light scattering setup to measure hydrodynamic size, polydispersity, and the diffusion coefficient for each anti-IL-8 mAb mutant. Data were analysed using the Lunatic & Stunner Client software (version 8.1.0.254). The measurement temperature was set as 25 ℃ and five, 10-second measurements were acquired with a corresponding 1 % extinction coefficient of 1.55 AU*L/(g*cm) for all samples. Custom dispersant settings were applied (viscosity 1.26 cP and refractive index 1.33 at 20 °C) and all mAbs were prepared in formulation buffer (0.5–20 mg/mL) for WT and mutant variants. The Lunatic & Stunner software (v8.1.0.244) were used for data export, and corresponding diffusion coefficients were used to calculate interaction parameters (k_D_) using linear regression plots (Eq. 5).

(5) $$D_{\mathrm{app}}=D_{0}\left(1+k_{D}c\right)$$

Where D_app_ refers to the apparent diffusion coefficient, D_0_ the self-diffusion coefficient at infinite dilution, and k_D_ the interaction parameter.

#### 4.3.5 Analysis of anti-IL-8 mAb charge distribution profile

We used the iCE3 capillary isoelectric focusing instrument with a PrinCE autosampler (Protein Simple) to measure charge distribution profiles. A range of pI markers (pI 3.85–8.77) were used to capture all main and impurity isoforms for each sample (Bio-Teche, Protein Simple, USA). To minimise self-association, we used 2 M urea and ampholytes (Bio-Teche, Protein Simple, USA) in the pH 3.0–10.0 and 8.0–10.5 ranges at a 1:1 ratio in the buffer mix. All samples were diluted to 1 mg/mL in deionised water prior to a final dilution to 0.4 mg/mL in analyte buffer. The iCE3 instrument was set to the following parameters: a pre-focus voltage of 1500 V; a 10–12-minute focus voltage of 3000 V; an autosampler and transfer capillary temperature of 15 °C; UV detection at 280 nm; a sample injection pressure of 2000 mbar; a pre-focus time of 1 min; and a focus time of 10–12 min. All data were imported to the Empower 3 software (v4, Waters, US) for analysis.

#### 4.3.6 Analysis of anti-IL-8 mAb self-interaction

We used Affinity-Capture Self-Interaction Nanoparticle Spectroscopy (AC-SINS) to assess self-association propensity in the anti-IL-8 mAb panel. [45] Goat anti-human Fc and whole goat IgG antibodies (Jackson ImmunoResearch, PA, USA) were prepared in 20 mM acetate buffer (pH 4.3) and diluted to achieve final concentrations of 320 µg anti-Fc IgG and 80 µg goat whole IgG, then mixed with 20 nM colloidal gold nanoparticle suspension (Ted Pella Inc., CA, USA, concentration 7.0×10^11^ particles /mL). After incubation and centrifugation, anti-IL-8 mAb test samples were prepared at 50 μg/mL in phosphate-buffered saline (Gibco, Thermo Fisher Scientific, MA, USA). Aliquots (99 μL) of each sample were added to wells of a 96-well plate, with 11 μL of gold nanoparticle suspension added to each well, resulting in a final solution concentration of 50 µg/mL test mAb, 10x bead:anti-Fc conjugate and 0.02 mg/mL PEG2000. All samples were mixed, incubated for 90 min and gently centrifuged to remove air bubbles. Following incubation, the absorbance spectra (450–650 nm) of the antibody-gold conjugates and analysed using a Pherastar FSX (BMG Labtech Ltd., Germany) plate reader. The spectra were analysed with MARS software (v3.32, BMG Labtech Ltd., Germany), applying smoothing to the best fit curves and the difference in plasmon wavelengths for each sample was calculated. Experimental cutoffs included a < 535 nm wavelength for negative controls (i.e*.,* buffer), and a red shift of > 10 nm was flagged as a candidate at high risk of self-association.

#### 4.3.7 Analysis of unfolding temperatures

Thermal differential scanning fluorimetry (DSF) measurements were performed using a Prometheus NT.48 (NanoTemper Technologies, Germany) equipped with back-reflection technology for high-throughput analysis of unfolding temperature (T_m_), calculated from the intrinsic fluorescence intensity ratio of tyrosine and tryptophan (350/330 nm). [46] Prior to each experiment, the excitation power was set to achieve ≥ 5000 counts in the discovery scan. Corresponding profiles were analysed in Prometheus NT.48 and the first derivative calculated. A temperature ramp of 2 °C/minute from 20–95 °C was performed for each set of capillaries. Drop lines were assessed and corrected, to determine first-derivative peaks, marking the unfolding temperatures of antibody domains (T_m1_ to T_m3_) and the unfolding onset (T_onset_). The first derivative peak of the scattering profile marked the aggregation temperature (T_agg_) values.

#### 4.3.8 Viscosity measurement

Viscosity curves were generated using the VROC Initium (Rheosense, United States). The protocol was optimised to measure viscosity samples using the ‘Auto’ shear rate function and fixed shear rates ranging from 100–2000 s^−1^. The resulting data were filtered based on specific criteria, including the exclusion of priming segments, ensuring the percent full scale fell within the 5–95 % range, maintaining an R^2^ fit of the pressure sensor position of ≥ 0.998, and steady plateaus with no drift in transient curves. Exponential-growth decay fits were applied to each viscosity-concentration curve, with the equation;

(6) $${\eta =Y_{0}e}^{\mathrm{kC}}$$

Where η is the dynamic viscosity (cP), Y_0_ the intercept (cP), k the rate constant (mg/mL^−1^), and c is the concentration (mg/mL.

**Statistical approaches*****.*** GraphPad Prism (v5.04) was used for plotting scatter plots and bar graphs, and ANOVA statistical analysis to determine significant differences in experimental data. JMP Pro (v16.0.0, 2021) was used for the multivariate analyses of computational and experimental data to establish existing correlations.

## Author statement

All authors have read and approve the submission of this manuscript to the journal of computational and structural biotechnology. GBA, VS, MP, PS, RC, WL are employees of GlaxoSmithKline. All other authors declare no conflict of interest.

## CRediT authorship contribution statement

**Glenn A Burley:** Writing – review & editing, Writing – original draft, Funding acquisition. **Craig Jamieson:** Writing – review & editing, Supervision. **Ricky Casey:** Writing – review & editing, Supervision, Conceptualization. **Mitul Patel:** Methodology, Investigation. **Zahra Rattray:** Writing – review & editing, Writing – original draft, Visualization, Supervision, Funding acquisition, Formal analysis, Conceptualization. **William Lewis:** Writing – review & editing, Validation, Supervision, Funding acquisition, Conceptualization. **Georgina B Armstrong:** Writing – review & editing, Writing – original draft, Methodology, Investigation, Formal analysis, Data curation. **Paula Sanches:** Methodology, Investigation. **Vidhi Shah:** Writing – review & editing, Methodology, Investigation, Formal analysis.

## Declaration of Competing Interest

Georgina B Armstrong, Vidhi Shah, Paula Sanches,Mitul Patel, Ricky Casey, and William Lewis are employees of GlaxoSmithKline. The remainder of the authors declare no competing interests.

## Data Availability

All data required to study the conclusions of this article, are included in supplementary files. The authors declare that all data needed to support the findings of this study are presented in the body of this article and the supplementary information. Additional details are available from the corresponding authors upon request.
