# Supplementary material for: A framework for the biophysical screening of antibody mutations targeting solvent-accessible hydrophobic and electrostatic patches for enhanced viscosity profiles
Source: Comput Struct Biotechnol J. 2024 May 24;23:2345–57. doi: 10.1016/j.csbj.2024.05.041 (PMC11167247; doi:10.1016/j.csbj.2024.05.041)
Supplement: Supplementary file 1 — Supplementary material [file mmc1.docx]

**Patch analysis of candidate mutants**

Top-scoring residues contributing to hydrophobic (*res_hyd*), positive (*res_pos*) and negative (*res_neg*) patches. Residues close to interactions with the GroBeta ligand are marked with an asterisk.

| **res_hyd score (Å2)** | **Residue** | **Position** | **Mutant variant** |
| --- | --- | --- | --- |
| 61.4 | F83 | Framework L | F83Q |
| 57.1 | Y55* | CDRH2 | Y55L |
| 46.9 | L110 | Framework H | L110Q |
| 44.7 | F57* | CDRH2 | F57L |
| 40 | Y99 | CDRH3 | Y99L |
| 33 | V11 | Framework H | V11Q |
| 32.9 | V5 | Framework H | V5Q |
| 28 | W32 | CDRH2 | W32Q |
| **res_pos score (Å2)** | **Residue** | **Position** | **Mutant variant** |
| 52.9 | R53 | CRDL2 | R53G |
| 44.6 | K42 | Framework L | K42E |
| 41.8 | K23 | Framework H | K23E |
| 35.3 | K63 | CDRH2 | K63E |
| 31.8 | R18 | Framework L | R18G |
| 31.3 | K13 | Framework H | K13E |
| 26.3 | R85 | Framework H | R85G |
| 24.8 | R70 | Framework H | R70G |
| **res_neg score (Å2)** | **Residue** | **Position** | **Mutant variant** |
| 70.5 | E30A* | CDRL1 | E30AQ |
| 38.9 | D56 | CDRL2 | D56N |
| 27.1 | Q27 | CDRL1 | Q27N |
| 24.2 | D70 | Framework L | D70N |
| 23.6 | D28 | CDRL1 | D28N |
| 20.3 | E10 | Framework H | E10Q |
| 20.3 | E87 | Framework H | E97Q |
| 18.1 | D17 | Framework L | D17N |

**Patch numbers and corresponding surface area for mAb1 WT and the generated mutants**

Table S2 Patch numbers and corresponding area coverage for candidate mutant Fv homology constructs.

| Position of mutation | Molecule | patch_ hyd (Å^2^) | patch_ hyd_n | patch_ ion (Å^2^) | patch_ ion_n | patch_ pos (Å^2^) | patch_ pos_n | patch_ neg (Å^2^) | patch_ neg_n | patch_ cdr_pos (Å^2^) | patch_ cdr_pos_n | patch_ cdr_neg (Å^2^) | patch_ cdr_neg_n | patch_ cdr_hyd (Å^2^) | patch_ cdr_hyd_n | Res_ASA (Å^2^) | BSA_ LC_HC |
| --- | --- | --- | --- | --- | --- | --- | --- | --- | --- | --- | --- | --- | --- | --- | --- | --- | --- |
| - | WT | 680 | 9 | 1100 | 23 | 690 | 14 | 410 | 9 | 380 | 6 | 280 | 5 | 280 | 2 | 10078.9 | 681.10 |
| FWR L | D17N | 620 | 8 | 1190 | 23 | 760 | 14 | 430 | 9 | 380 | 6 | 300 | 5 | 260 | 2 | 10109.3 | 681.19 |
| FWR L | D70N | 660 | 9 | 1071 | 22 | 690 | 14 | 380 | 8 | 380 | 6 | 250 | 4 | 260 | 2 | 10051.4 | 681.19 |
| FWR L | F83Q | 540 | 8 | 1190 | 24 | 740 | 14 | 450 | 10 | 430 | 6 | 290 | 5 | 260 | 2 | 10080.5 | 681.19 |
| FWR L | R18G | 660 | 9 | 1080 | 22 | 660 | 13 | 420 | 9 | 380 | 6 | 290 | 5 | 260 | 2 | 10036.4 | 681.19 |
| FWR L | K42E | 640 | 8 | 1060 | 22 | 640 | 13 | 420 | 9 | 380 | 6 | 290 | 5 | 260 | 2 | 10016 | 683.39 |
| FWR H | V5Q | 620 | 8 | 1170 | 24 | 700 | 14 | 470 | 10 | 380 | 6 | 290 | 5 | 260 | 2 | 10060.9 | 680.64 |
| FWR H | E10Q | 660 | 9 | 1140 | 23 | 740 | 15 | 400 | 8 | 380 | 6 | 300 | 5 | 260 | 2 | 10069.2 | 681.19 |
| FWR H | E87Q | 660 | 9 | 1130 | 22 | 740 | 14 | 390 | 8 | 380 | 6 | 290 | 5 | 260 | 2 | 10075.7 | 681.19 |
| FWR H | L110Q | 590 | 8 | 1110 | 23 | 690 | 14 | 420 | 9 | 380 | 6 | 290 | 5 | 260 | 2 | 10058.1 | 681.14 |
| FWR H | V11Q | 620 | 8 | 1120 | 23 | 690 | 14 | 430 | 9 | 380 | 6 | 300 | 5 | 260 | 2 | 10073.1 | 681.19 |
| FWR H | R85G | 660 | 9 | 1070 | 21 | 650 | 13 | 420 | 8 | 380 | 6 | 290 | 5 | 260 | 2 | 10086.4 | 681.19 |
| FWR H | R70G | 630 | 8 | 1080 | 22 | 650 | 13 | 430 | 9 | 340 | 5 | 290 | 5 | 260 | 2 | 10136.5 | 681.19 |
| FWR H | K23E | 620 | 8 | 1140 | 23 | 640 | 13 | 500 | 10 | 380 | 6 | 290 | 5 | 260 | 2 | 10090 | 681.19 |
| FWR H | K13E | 660 | 9 | 1160 | 22 | 660 | 13 | 500 | 9 | 380 | 6 | 290 | 5 | 260 | 2 | 10096.7 | 681.19 |
| CRDL2 | R53G | 710 | 9 | 1030 | 22 | 630 | 13 | 400 | 9 | 320 | 5 | 270 | 5 | 310 | 2 | 10057.6 | 661.34 |
| CDRL2 | D56N | 640 | 8 | 1120 | 22 | 740 | 14 | 380 | 8 | 350 | 5 | 250 | 4 | 270 | 2 | 10032.1 | 702.68 |
| CDRL1 | D28N | 700 | 9 | 1040 | 24 | 690 | 14 | 350 | 10 | 380 | 6 | 220 | 6 | 300 | 2 | 10050.4 | 681.19 |
| CDRL1 | E30aQ | 700 | 9 | 1090 | 24 | 690 | 14 | 400 | 10 | 380 | 6 | 270 | 6 | 300 | 2 | 10038.9 | 684.97 |
| CDRL1 | Q27N | 660 | 9 | 1170 | 22 | 680 | 14 | 490 | 8 | 370 | 6 | 360 | 4 | 260 | 2 | 10055.5 | 681.19 |
| CDRH3 | W105Q | 630 | 8 | 1170 | 24 | 720 | 14 | 450 | 10 | 340 | 5 | 320 | 6 | 260 | 2 | 10096.4 | 682.89 |
| CDRH3 | Y99L | 670 | 9 | 1130 | 23 | 700 | 14 | 430 | 9 | 390 | 6 | 300 | 5 | 270 | 2 | 10072.1 | 661.51 |
| CDRH3 | W102bQ | 670 | 9 | 1110 | 24 | 680 | 14 | 430 | 10 | 370 | 6 | 300 | 6 | 270 | 2 | 10064.5 | 608.28 |
| CDRH2 | W32Q | 610 | 11 | 1120 | 23 | 700 | 14 | 420 | 9 | 390 | 6 | 290 | 5 | 180 | 3 | 9997.4 | 688.06 |
| CDRH2 | F57L | 660 | 9 | 1090 | 23 | 670 | 14 | 420 | 9 | 360 | 6 | 290 | 5 | 260 | 2 | 9999.2 | 697.78 |
| CDRH2 | Y55L | 660 | 9 | 1060 | 22 | 640 | 13 | 420 | 9 | 330 | 5 | 290 | 5 | 260 | 2 | 10036.7 | 678.32 |
| CDRH2 | K63E | 660 | 9 | 1160 | 23 | 640 | 13 | 520 | 10 | 340 | 5 | 390 | 6 | 260 | 2 | 10105.9 | 684.52 |
