# Supplementary material for: A framework for the biophysical screening of antibody mutations targeting solvent-accessible hydrophobic and electrostatic patches for enhanced viscosity profiles
Source: Comput Struct Biotechnol J. 2024 May 24;23:2345–57. doi: 10.1016/j.csbj.2024.05.041 (PMC11167247; doi:10.1016/j.csbj.2024.05.041)
Supplement: Supplementary file 2 — Supplementary material [file mmc2.docx]

Physicochemical molecular descriptors computed for WT and mutant homology models that have been used in previous studies to predict viscosity.

| **Name** | **Description** |
| --- | --- |
| **patch_hyd** | Summed area of hydrophobic patches (Å^2^).1 |
| **patch_hyd_n** | Summed number of hydrophobic patches.^1^ |
| **patch_pos Å2** | Summed area of positive patches (Å^2^).^1^ |
| **patch_pos_n** | Summed number of positive patches.^1^ |
| **patch_neg Å2** | Summed area of negative patches (Å^2^). ^1^ |
| **patch_neg_n** | Summed number of negative patches.^1^ |
| **patch_ion** | Summed area of ionic (positive and negative) patches (Å^2^).^1^ |
| **patch_ion_n** | Summed number of charged (positive and negative) patches. ^1^ |
| **asa_hyd Å2** | Solvent-accessible surface area of hydrophobic atoms of a protein (Å^2^).^1^ |
| **patch_cdr_hyd** | Summed area of hydrophobic patches near the CDRs (Å^2^).^1^ |
| **patch_cdr_hyd_n** | Summed number of hydrophobic patches near the CDRs. ^1^ |
| **patch_cdr_pos** | Summed area of positive patches near the CDRs (Å^2^).^1^ |
| **patch_cdr_pos_n** | Summed number of positive patches near the CDRs. ^1^ |
| **patch_cdr_neg** | Summed area of negative patches near the CDRs (Å^2^).^1^ |
| **patch_cdr_neg_n** | Summed number of negative patches near the CDRs.^1^ |
| **Hydrophobic Imbalance** | A vector that describes the displacement of the superficial geometric centre of the protein when the respective ASA values of each amino acid is considered.^[[1]](#endnote-1)^ This was calculated through the descriptors function in the BioMOE module in MOE 2020. Default parameters were used with no sampling. This was calculated off the Fv model at default values of pH 7.4, temperature of 300K and a salt concentration of 0.1M.^3^ |
| **Fv_chml** | The Fv heavy chain (V_H_) charge – Fv light chain (V_L_) charge. This was calculated through the descriptors function in the BioMOE module in MOE 2020. Default parameters were used with no sampling. This was calculated from the Fv model at default values of pH 7.4, temperature of 300K and a salt concentration of 0.1M.^[[2]](#endnote-2)^ |
| **Pro_Fv_net_charge** | The protein net charge on Fv only. This was calculated through the descriptors function in the BioMOE module in MOE 2020. Default parameters were used with no sampling. This was calculated off the Fv model at default values of pH 7.4, temperature of 300K and a salt concentration of 0.1M.^3^ |
| **Pro_net_charge** | The protein net charge. This was calculated through the descriptors function in the BioMOE module in MOE 2020. Default parameters were used with no sampling. This was calculated off the Fv model at default values of pH 7.4, temperature of 300K and a salt concentration of 0.1M.^3^ |
| **Net_charge** | The formal protein net charge at a given pH. This was calculated through the Protein Properties tool in MOE 2020. The target pH was set to 6, temperature set to 300K and salt concentration to 0.1M.^1^ |
| **Dipole_moment** | Dipole calculated across the protein from uneven distribution of charges. This was calculated through the Protein Properties tool in MOE 2020. The target pH was set to 6, temperature set to 300K and salt concentration to 0.1M.^1^ |
| **Hyd_moment** | Hydrophobicity moment where each residue side chain hydrophobicity is calculated from the Kyte-Doolittle scale across the length of the protein. ^[[3]](#endnote-3)^ This was calculated through the Protein Properties tool in MOE 2020. The target pH was set to 6, temperature set to 300K and salt concentration to 0.1M.^1^ |
| **Hydrophobicity Index** | The summation of hydrophobic residues’ Eisenberg scores over the summation of hydrophilic residues’ Eisenberg scores. Sharma et al. correlated Lower Eisenberg scores with lower viscosity.^[[4]](#endnote-4)^ |
| **Zeta** | Zeta potential is the electrical potential observed at the slipping plane. This was calculated through the Protein Properties tool in MOE 2020. The target pH was set to 6, temperature set to 300K and salt concentration to 0.1M.^1,^ ^[[5]](#endnote-5)^ |
| **pI_seq** | The isoelectric point of a protein calculated from amino acid composition. This was calculated through the Protein Properties tool in MOE 2020. The target pH was set to 6, temperature set to 300K and salt concentration to 0.1M. ^1,^ ^[[6]](#endnote-6)^ |
| **BSA_LC_HC** | The buried surface area (BSA) between the heavy and light chains in Å^2^. This was calculated through the descriptors function in the BioMOE module in MOE 2020. Default parameters were used with no sampling. This was calculated off This was calculated off the Fv model at default values of pH 7.4, temperature of 300K and a salt concentration of 0.1M.^3^ |
| **Pro_hyd_moment** | Hydrophobicity moment where each residue side chain hydrophobicity is calculated from the Kyte-Doolittle scale across the length of the protein. This was calculated through the descriptors function in the BioMOE module in MOE 2020. Default parameters were used with no sampling. This was calculated off the Fv model at default values of pH 7.4, temperature of 300K and a salt concentration of 0.1M. ^3, 4^ |
| **Ens_charge** | The ensemble average charge of the full molecule. This was calculated through the Protein Properties tool in MOE 2020. The target pH was set to 6, temperature set to 300K and salt concentration to 0.1M.^1^ |
| **pI_3D** | The isoelectric point of the molecule calculated through a modified version of Sillero’s model. The PROPKA algorithm is used. This was calculated through the Protein Properties tool in MOE 2020. The target pH was set to 6, temperature set to 300K and salt concentration to 0.1M.^1^ |
| **Fv charge symmetry (FvSCP)** | Charge symmetry of the Fv was calculated with charge of the light chain multiplied by the net charge of the heavy chain.^5^ |
| **Res_ASA** | The summed contribution from each residue to the accessible surface area in Å^2^. This was calculated through the Protein Properties tool in MOE 2020 and manually summed subsequently. The target pH was set to 6, temperature set to 300K and salt concentration to 0.1M.^1^ |
| **Res_hyd** | The summed hydrophobic contribution from each residue to hydrophobic patch area in Å^2^. This was calculated through the Protein Properties tool in MOE 2020 and manually summed subsequently. The target pH was set to 6, temperature set to 300K and salt concentration to 0.1M.^1^ |
| **Dipole moment/hyd moment ratio** | The ratio of Fv dipole moment over the Fv hydrophobic moment to describe the balance of polar versus nonpolar distributions per molecule. This was previously identified as an intrinsic non-redundant descriptor for a dataset of commercial mAbs.^[[7]](#endnote-7)^ |
| **Ionic/hydrophobic patch area ratio** | The ratio of Fv ionic patch area to hydrophobic patch area. This was previously identified as an intrinsic non-redundant descriptor for a dataset of commercial mAbs.^7^ |

**Physicochemical molecular descriptor results of mutant variants.**

Charge-based physicochemical descriptors computed for each mAb1 mutant Fv homology construct.

| Position of mutation | Molecule | Fv_chml | pro_Fv_net_charge | pro_net_charge | net_ charge | dipole_moment | Predicted zeta at Deybe length (mV) | pI_seq | ens_ charge | pI_3D | VL net charge | VH net charge | Fv charge symmetry | Deep SCM |
| --- | --- | --- | --- | --- | --- | --- | --- | --- | --- | --- | --- | --- | --- | --- |
| - | WT | 3 | 3.0 | -0.41 | 0.05 | 554.21 | 0.19 | 6.42 | 2.01 | 6.23 | -1.23 | 3.93 | -4.83 | 1197.42 |
| FWR L | D17N | 2 | 4.0 | 0.17 | 0.62 | 578.20 | 1.79 | 6.68 | 3.30 | 7.61 | -0.32 | 3.93 | -1.26 | 1164.43 |
| FWR L | D70N | 2 | 4.0 | 0.17 | 0.63 | 466.75 | 1.58 | 6.68 | 2.90 | 7.61 | -0.32 | 3.93 | -1.26 | 1136.54 |
| FWR L | F83Q | 3 | 3.0 | -0.40 | 0.05 | 554.97 | 0.19 | 6.42 | 2.10 | 6.23 | -1.23 | 3.93 | -4.83 | 1203.71 |
| FWR L | R18G | 4 | 2.0 | -1.29 | -0.84 | 602.97 | -2.18 | 6.07 | 1.31 | 4.92 | -2.18 | 3.91 | -8.52 | 1224.63 |
| FWR L | K42E | 5 | 1.0 | -2.27 | -1.81 | 482.48 | -4.14 | 5.62 | 0.07 | 4.56 | -3.16 | 3.93 | -12.42 | 1253.89 |
| FWR H | V5Q | 3 | 3.0 | -0.41 | 0.05 | 561.51 | 0.19 | 6.42 | 2.25 | 6.23 | -1.23 | 3.83 | -4.71 | 1194.19 |
| FWR H | E10Q | 4 | 4.0 | 0.17 | 0.62 | 614.54 | 1.64 | 6.68 | 3.00 | 7.61 | -1.23 | 4.83 | -5.94 | 1162.09 |
| FWR H | E87Q | 4 | 4.0 | 0.17 | 0.63 | 528.25 | 1.54 | 6.68 | 3.25 | 7.61 | -1.23 | 4.88 | -6.00 | 1178.73 |
| FWR H | L110Q | 3 | 3.0 | -0.41 | 0.05 | 555.58 | 0.19 | 6.42 | 1.96 | 6.23 | -1.23 | 3.91 | -4.81 | 1197.28 |
| FWR H | V11Q | 3 | 3.0 | -0.41 | 0.05 | 557.56 | 0.19 | 6.42 | 2.10 | 6.23 | -1.23 | 3.92 | -4.82 | 1206.02 |
| FWR H | R85G | 2 | 2.0 | -1.30 | -0.84 | 587.77 | -1.98 | 6.07 | 0.98 | 4.92 | -1.23 | 2.93 | -3.60 | 1217.96 |
| FWR H | R70G | 2 | 2.0 | -1.29 | -0.83 | 565.14 | -1.99 | 6.07 | 0.94 | 4.94 | -1.23 | 2.93 | -3.60 | 1261.49 |
| FWR H | K23E | 1 | 1.0 | -2.21 | -1.72 | 390.65 | -4.03 | 5.62 | 0.11 | 4.68 | -1.23 | 0.95 | -1.17 | 1317.79 |
| FWR H | K13E | 1 | 1.0 | -2.21 | -1.73 | 578.86 | -4.40 | 5.62 | 0.25 | 4.64 | -1.23 | 0.94 | -1.16 | 1290.93 |
| CRDL2 | R53G | 4 | 2.0 | -1.29 | -0.83 | 576.62 | -1.96 | 6.07 | 1.01 | 4.93 | -2.18 | 3.93 | -8.57 | 1256.43 |
| CDRL2 | D56N | 2 | 4.0 | 0.17 | 0.63 | 598.31 | 1.51 | 6.68 | 3.00 | 7.61 | -0.32 | 3.82 | -1.22 | 1136.34 |
| CDRL1 | D28N | 2 | 4.0 | 0.16 | 0.62 | 484.86 | 1.46 | 6.68 | 2.88 | 7.58 | -0.34 | 3.93 | -1.34 | 1062.51 |
| CDRL1 | E30aQ | 2 | 4.0 | 0.00 | 0.71 | 511.01 | 1.67 | 6.68 | 2.96 | 7.40 | -0.35 | 3.93 | -1.38 | 1104.96 |
| CDRL1 | Q27N | 3 | 3.0 | -0.47 | 0.05 | 557.86 | 0.20 | 6.42 | 2.11 | 6.23 | -1.23 | 3.93 | -4.83 | 1194.81 |
| CDRH3 | W105Q | 3 | 3.0 | -0.41 | 0.06 | 547.28 | 0.21 | 6.42 | 2.62 | 6.25 | -1.23 | 3.94 | -4.85 | 1188.64 |
| CDRH3 | Y99L | 3 | 3.0 | -0.40 | 0.05 | 551.36 | 0.19 | 6.42 | 1.92 | 6.23 | -1.23 | 3.9 | -4.80 | 1189.43 |
| CDRH3 | W102bQ | 3 | 3.0 | -0.41 | 0.06 | 552.14 | 0.20 | 6.42 | 2.17 | 6.24 | -1.33 | 3.87 | -5.15 | 1190.74 |
| CDRH2 | W32Q | 3 | 3.0 | -0.41 | 0.05 | 566.44 | 0.21 | 6.42 | 2.38 | 6.24 | -1.22 | 3.92 | -4.78 | 1213.87 |
| CDRH2 | F57L | 3 | 3.0 | -0.45 | 0.05 | 555.44 | 0.19 | 6.42 | 2.07 | 6.21 | -1.23 | 3.93 | -4.83 | 1199.18 |
| CDRH2 | Y55L | 3 | 3.0 | -0.41 | 0.05 | 555.09 | 0.19 | 6.42 | 2.11 | 6.23 | -1.23 | 3.93 | -4.83 | 1200.79 |
| CDRH2 | K63E | 1 | 1.0 | -2.19 | -1.71 | 655.50 | -4.03 | 5.62 | 0.13 | 4.65 | -1.23 | 0.94 | -1.16 | 1279.49 |

Hydrophobicity-based physicochemical molecular descriptors and TANGO aggregation propensity scores of mAb1 mutant variants. Dipole and ionic to hydrophobicity ratios are also reported.

| Position of mutation | Molecule | Hydrophobic imbalance | hyd_moment | pro_hyd_moment | Hydrophobic index | Normalised hydrophobicity score (%) | ASA_hyd Å^2^ | Res_hyd Å^2^ | Dipole moment/hyd patch area | Ionic/ hydrophobic patch area ratio | TANGO Aggregation propensity |
| --- | --- | --- | --- | --- | --- | --- | --- | --- | --- | --- | --- |
| - | WT | 1.08 | 396.57 | 396.57 | 1.094 | 5.14 | 5647.53 | 518.50 | 1.07 | 1.62 | 1603.94 |
| FWR L | D17N | 1.14 | 395.70 | 395.70 | 1.096 | 4.91 | 5581.12 | 496 | 1.20 | 1.92 | 1590.12 |
| FWR L | D70N | 1.09 | 397.58 | 397.58 | 1.096 | 4.96 | 5648.77 | 498.3 | 0.95 | 1.62 | 1627.07 |
| FWR L | F83Q | 1.22 | 334.44 | 334.44 | 1.065 | 4.05 | 5575.46 | 408.2 | 1.39 | 2.20 | 1577.65 |
| FWR L | R18G | 1.03 | 432.39 | 432.39 | 1.140 | 4.96 | 5639.26 | 498.1 | 1.23 | 1.64 | 1589.75 |
| FWR L | K42E | 1.13 | 400.56 | 400.56 | 1.105 | 5.06 | 5574.70 | 507.3 | 0.96 | 1.66 | 1602.38 |
| FWR H | V5Q | 0.80 | 325.88 | 325.88 | 1.067 | 4.96 | 5599.16 | 498.8 | 1.15 | 1.89 | 1603.94 |
| FWR H | E10Q | 1.08 | 396.95 | 396.95 | 1.092 | 4.96 | 5662.86 | 499.1 | 1.25 | 1.73 | 1602.71 |
| FWR H | E87Q | 1.09 | 396.28 | 396.28 | 1.092 | 4.94 | 5664.91 | 498 | 1.08 | 1.71 | 1632.24 |
| FWR H | L110Q | 1.03 | 355.72 | 355.72 | 1.067 | 4.22 | 5604.93 | 424.2 | 1.32 | 1.88 | 1530.25 |
| FWR H | V11Q | 1.08 | 378.72 | 378.72 | 1.067 | 4.95 | 5598.55 | 498.6 | 1.14 | 1.81 | 1603.94 |
| FWR H | R85G | 1.07 | 394.59 | 394.59 | 1.140 | 4.94 | 5647.25 | 498.4 | 1.20 | 1.62 | 1603.62 |
| FWR H | R70G | 1.12 | 401.40 | 401.40 | 1.140 | 4.92 | 5666.67 | 498.8 | 1.15 | 1.71 | 1603.94 |
| FWR H | K23E | 1.08 | 399.60 | 399.60 | 1.105 | 4.95 | 5557.77 | 499.4 | 0.80 | 1.84 | 1624.98 |
| FWR H | K13E | 1.08 | 396.35 | 396.35 | 1.105 | 4.94 | 5627.99 | 499.1 | 1.16 | 1.76 | 1605.15 |
| CRDL2 | R53G | 1.08 | 401.05 | 401.05 | 1.140 | 5.40 | 5691.33 | 542.8 | 1.07 | 1.45 | 1897.13 |
| CDRL2 | D56N | 1.11 | 397.34 | 397.34 | 1.096 | 5.02 | 5649.88 | 503.3 | 1.20 | 1.75 | 1602.95 |
| CDRL1 | D28N | 1.05 | 396.57 | 396.57 | 1.096 | 5.32 | 5652.63 | 535 | 0.91 | 1.49 | 1603.43 |
| CDRL1 | E30aQ | 1.06 | 397.01 | 397.01 | 1.092 | 5.37 | 5655.14 | 538.6 | 0.96 | 1.56 | 1638 |
| CDRL1 | Q27N | 1.10 | 396.47 | 396.47 | 1.095 | 4.98 | 5618.84 | 500.3 | 1.12 | 1.77 | 1603.79 |
| CDRH3 | W105Q | 1.03 | 380.86 | 380.86 | 1.070 | 4.94 | 5599.76 | 498.6 | 1.09 | 1.86 | 1603.88 |
| CDRH3 | Y99L | 1.09 | 393.52 | 393.52 | 1.105 | 5.11 | 5675.74 | 514.2 | 1.08 | 1.69 | 1603.96 |
| CDRH3 | W102bQ | 1.04 | 402.35 | 402.35 | 1.070 | 4.97 | 5598.06 | 500.3 | 1.10 | 1.66 | 1604.05 |
| CDRH2 | W32Q | 1.11 | 408.27 | 408.27 | 1.070 | 3.44 | 5597.72 | 343.7 | 1.67 | 1.84 | 1357.71 |
| CDRH2 | F57L | 1.13 | 387.53 | 387.53 | 1.092 | 4.91 | 5663.06 | 491.3 | 1.13 | 1.65 | 1602.02 |
| CDRH2 | Y55L | 1.11 | 353.42 | 353.42 | 1.105 | 4.98 | 5664.70 | 500.3 | 1.13 | 1.61 | 1605.53 |
| CDRH2 | K63E | 1.08 | 392.53 | 392.53 | 1.105 | 4.93 | 5608.40 | 498.3 | 1.34 | 1.76 | 1605.17 |

1. . Salgado, J. C., Rapaport, I. & Asenjo, J. A. Predicting the behaviour of proteins in hydrophobic interaction chromatography: 1: Using the hydrophobic imbalance (HI) to describe their surface amino acid distribution. J. Chromatogr. A 1107, 110–119 (2006). [↑](#endnote-ref-1)
2. . Long, W. Bio-MOE: Custom MOE Biologics Applications. (2021). [↑](#endnote-ref-2)
3. . Kyte, J. & Doolittle, R. F. A simple method for displaying the hydropathic character of a protein. J. Mol. Biol. 157, 105–132 (1982). [↑](#endnote-ref-3)
4. . Sharma, V. K., et al. (2014). "In silico selection of therapeutic antibodies for develop ment: viscosity, clearance, and chemical stability." Proc Natl Acad Sci U S A 111(52): 18601-18606. [↑](#endnote-ref-4)
5. . Tanford, C. Physical chemistry of macromolecules. J. Pharm. Sci. 51, 190–190 (1962). [↑](#endnote-ref-5)
6. . Sillero, A. & Ribeiro, J. M. Isoelectric points of proteins: Theoretical determination. Anal. Biochem. 179, 319–325 (1989). [↑](#endnote-ref-6)
7. . Ahmed, L. et al. Intrinsic physicochemical profile of marketed antibody-based biotherapeutics. Proc. Natl. Acad. Sci. 118, e2020577118 (2021). [↑](#endnote-ref-7)
