# Supplementary material for: A framework for the biophysical screening of antibody mutations targeting solvent-accessible hydrophobic and electrostatic patches for enhanced viscosity profiles
Source: Comput Struct Biotechnol J. 2024 May 24;23:2345–57. doi: 10.1016/j.csbj.2024.05.041 (PMC11167247; doi:10.1016/j.csbj.2024.05.041)
Supplement: Supplementary file 3 — Supplementary material [file mmc3.docx]

**Therapeutic antibody profiler (TAP) scores for mAb1 mutant variants**

***Therapeutic Antibody Profiler*** (<https://opig.stats.ox.ac.uk/webapps/sabdab-sabpred/sabpred/tap>). The therapeutic antibody profiler (TAP) is a developability ranking tool that incorporates CDR length, hydrophobicity, positive and negative charges of CDR patches and Fv charge symmetry of homology structures.^[[1]](#endnote-1)^ The web application was used to submit heavy and light chain sequences of the wild-type and mutant panel.


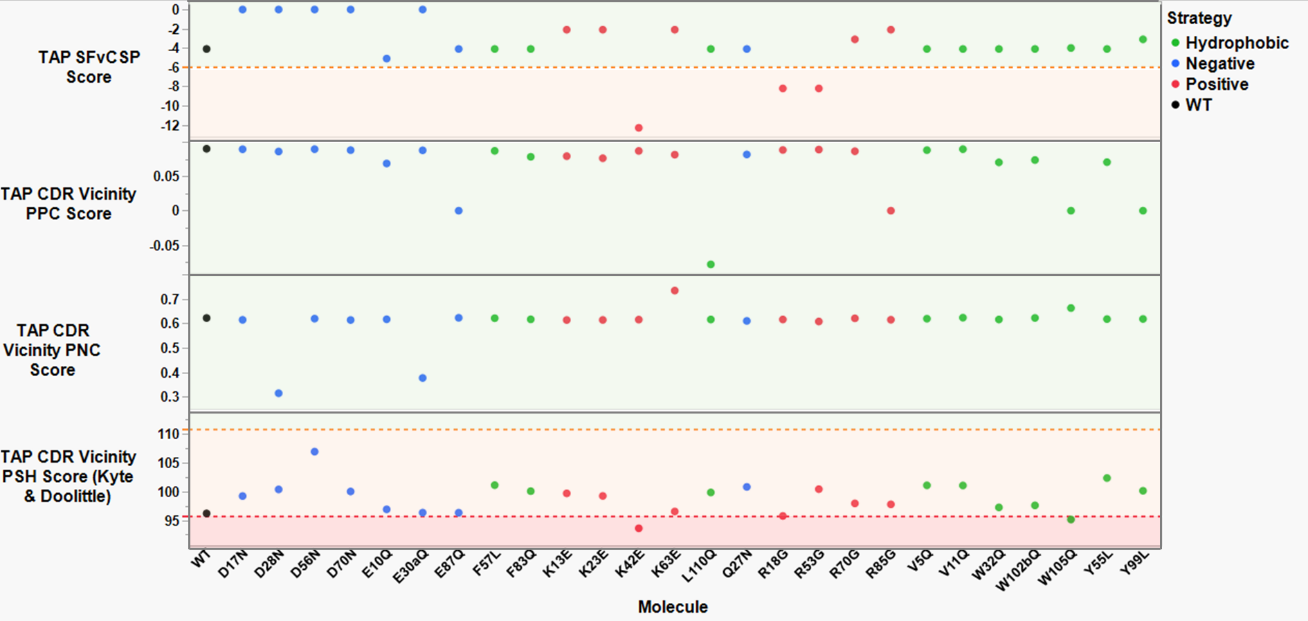


The Therapeutic Antibody Profiler (TAP) tool computed four structural attributes for mAb1 candidate mutants. This tool used the heavy and light chain sequences of the variable regions for each molecule and the ABodyBuilder2 tool was used to construct homology models. The red-amber-green thresholds were set from previous work analysing 137 clinical stage antibodies. For all mutants the CDR length was 46 residues which was within the green threshold. Structural Fv Charge Symmetry Parameter (SFvCSP) showed three positive-patch disrupting mutants with amber flags (K42E, R18G and R53G). The patches of positive charge (PPC) metric and the patches of negative charge (PNC) metric across the CDR vicinity showed no flags for all mutants. However, all mutants had at least an amber flag for the patches of Surface Hydrophobicity (PSH) metric across the CDR vicinity, with two red flags for K42E and W105Q.

1. . Thorsteinson, N., Gunn, J. R., Kelly, K., Long, W. & Labute, P. Structure-based charge calculations for predicting isoelectric point, viscosity, clearance, and profiling antibody therapeutics. MAbs 13, 1981805 (2021). [↑](#endnote-ref-1)
