# Supplementary material for: A framework for the biophysical screening of antibody mutations targeting solvent-accessible hydrophobic and electrostatic patches for enhanced viscosity profiles
Source: Comput Struct Biotechnol J. 2024 May 24;23:2345–57. doi: 10.1016/j.csbj.2024.05.041 (PMC11167247; doi:10.1016/j.csbj.2024.05.041)
Supplement: Supplementary file 4 — Supplementary material [file mmc4.docx]

**Modified Fv patch areas of expressed mutants**


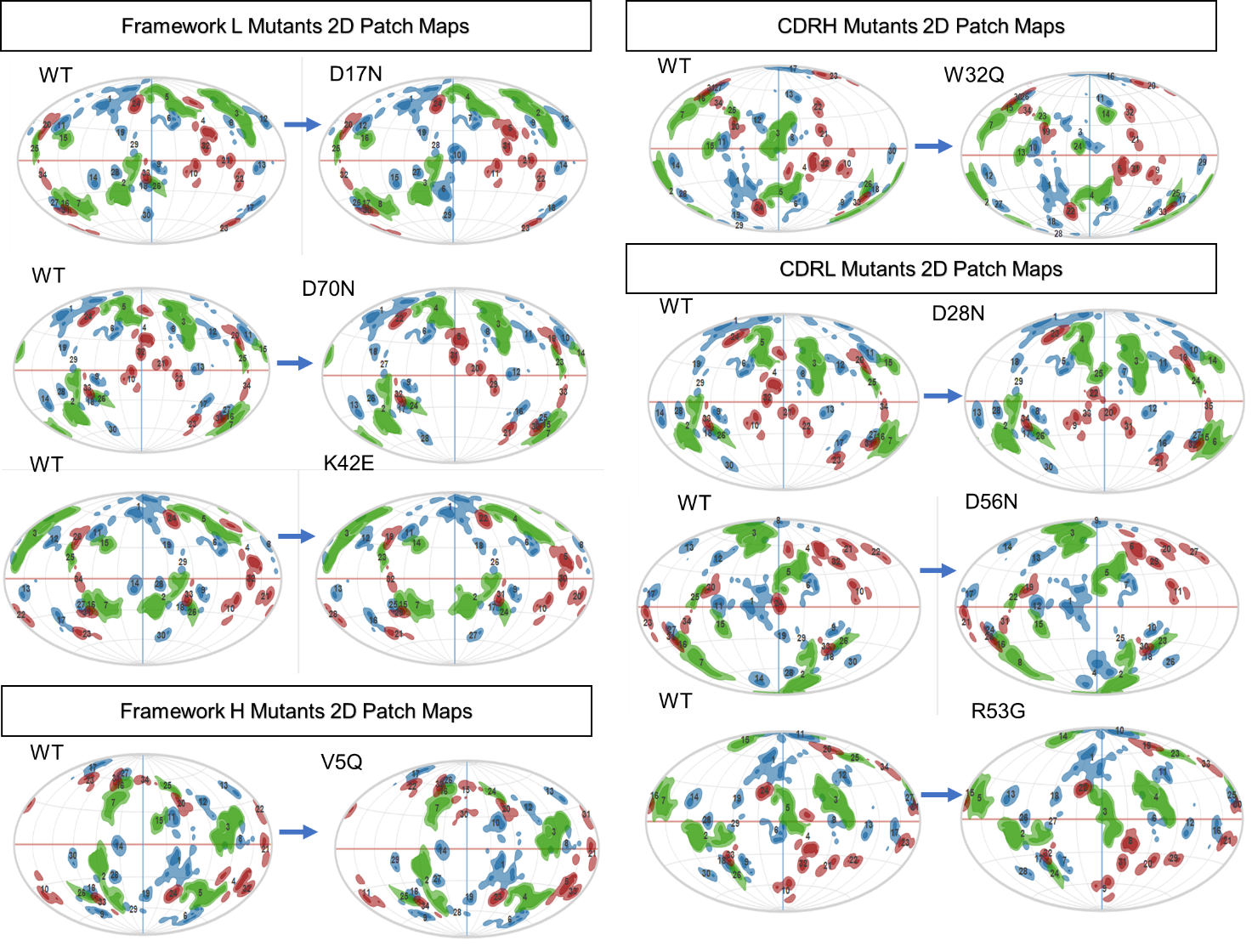


*Two-dimensional patch maps of expressed mutants Fv homology constructs*. Hydrophobic (green), positive (blue) and negative (red) patches were analysed for the area and energy changes for each expressed mAb1 mutant. The field of view is rotated for each WT and expressed mutant pair to have the site of mutation at the centre.

Quantitation of specific modified patch areas and energy changes for expressed mutants Fv homology constructs.

| **Molecule** | **WT 2D Map Number** | **Patch Type** | **Patch Area**  (Å^2^) | | **Average energy per Å^2^**  (kcal/mol) | | **Other significant residues** |
| --- | --- | --- | --- | --- | --- | --- | --- |
|  |  |  | **WT** | **Mutant** | **WT** | **Mutant** |  |
| **D17N** | 33 | neg | 30 | Removed | -49.42 | Removed | S14, G16 |
|  | 26 | hyd | 30 | Removed | -0.11 | Removed | P8, L11, A13, V19 |
| **D70N** | 10 | neg | 60 | Removed | -49.72 | Removed | Q24, Q69 |
| **K42E** | 14 | pos | 50 | Removed | -54.26 | Removed | P40, G41 |
|  | 28 | pos | 30 | Removed | -51.72 | Removed | K39, P40, E81, F83 |
| **W32Q** | 3 | hyd | 150 | 30 | -0.16 | -0.13 | Y51C |
|  | 8 | pos | 70 | 130 | -41.71 | -48.24 | H91, E93, S93A, P95 |
| **V5Q** | 15 | hyd | 40 | Removed | -0.14 | Removed | K23 |
|  | 11 | pos | 50 | 60 | -61.18 | -61.1 | K23 T74 S75 |
| **D28N** | 32 | neg | 30 | 30 | -55.96 | -43.85 | E30, G68 |
|  | N/A | hyd | 30 | Addition | -0.15 | Addition | E30A, Y32 |
| **D56N** | 24 | neg | 40 | Removed | -69.48 | Removed | N/A |
|  | 19 | pos | 40 | Removed | -45.54 | Removed | K45, G57, V58, P59 |
| **R53G** | 6 | pos | 80 | Removed | -53.93 | Removed | Y50 T52 L54 |
|  | 5 | hyd | 110 | 150 | -0.15 | -0.16 | Y32, Y49, Y50 |
