# Supplementary material for: A framework for the biophysical screening of antibody mutations targeting solvent-accessible hydrophobic and electrostatic patches for enhanced viscosity profiles
Source: Comput Struct Biotechnol J. 2024 May 24;23:2345–57. doi: 10.1016/j.csbj.2024.05.041 (PMC11167247; doi:10.1016/j.csbj.2024.05.041)
Supplement: Supplementary file 5 — Supplementary material [file mmc5.docx]

**Triage of candidate mutants**

Top and bottom scoring mAb1 mutants progressed to experimental characterisation based on min-max normalisation. Scoring was based on hydrophobic index, zeta potential, BSA_LC_HC, ens_charge, normalised hydrophobicity, and TANGO aggregation propensity. Each descriptor value was weighted evenly and normalised to ensure that the lower the score, the increased likelihood of reduced hypothesised viscosity.

| **Molecule** | **Mutation** | **Summed normalised score** |
| --- | --- | --- |
| WT (-) | - | 3.28 |
| W32Q (CDRH2) | Hydrophobic | 1.76 |
| D56N (CDRL2) | Negative | 2.13 |
| D17N (FWL) | Negative | 2.21 |
| D70N (FWL) | Negative | 2.35 |
| V5Q (FWH) | Hydrophobic | 2.37 |
| D28N (CDRL1) | Negative | 2.42 |
| R53G (CDRL2) | Positive | 5.83 |
| K42E (FWL) | Positive | 6.22 |
