# Supplementary material for: A framework for the biophysical screening of antibody mutations targeting solvent-accessible hydrophobic and electrostatic patches for enhanced viscosity profiles
Source: Comput Struct Biotechnol J. 2024 May 24;23:2345–57. doi: 10.1016/j.csbj.2024.05.041 (PMC11167247; doi:10.1016/j.csbj.2024.05.041)
Supplement: Supplementary file 6 — Supplementary material [file mmc6.docx]

# Supplementary figures and tables

**Biophysical Characterisation**

*Analysis of identity by mass spectrometry*

The sequence and composition of the anti-IL8 panel was verified using peptide fingerprinting mass spectrometry. 250μg of each sample was denatured with guanidine buffer (6M, pH 7.5), reduced with dithiothreitol (DTT 1M) and incubated for 20 minutes at ambient temperature. All samples were alkylated with 1M sodium iodoacetate and incubated for a further 30 minutes at ambient temperature and protected from light. A further reduction step was performed in DTT (1M), and the samples were desalted using Micro Bio-Spin 6 size exclusion columns (Bio-Rad, CA, USA). Samples were enzyme-digested with either trypsin or chymotrypsin (both sequencing-grade, Promega, WI, USA) at a 1:20 (w/w) ratio of chymotrypsin: mAb in a digestion buffer containing 50mM Tris, 1mM calcium chloride dihydrate (pH 7.5). Samples were incubated at 37 °C under agitation for two hours, prior to liquid chromatography-mass spectrometry (LC-MS) analysis with an Orbitrap Exploris™ 240 Mass Spectrometer (Thermo Fisher Scientific, MA, USA), controlled by Xcalibur software (version 4.4.16.14, Thermo Fisher Scientific, MA, USA). An ACQUITY UPLC PEPTIDE CSH C18 (Waters, US) 1.7 µm, 2.1 mm x 150 mm column was used for separating digested peptides with a column temperature of 40 °C. Mobile phase A was 0.1% Formic Acid LC-MS grade (Thermo Fisher Scientific, MA, USA) in LC-MS grade water and B was 0.1% Formic Acid in Acetonitrile LC-MS grade (Thermo Fisher Scientific, MA, USA). Step wise gradients were applied with 5-40 %B (over 80 min), 40-100 %B (5 min), plateau of 100 %B (5 min), and a return to 5 %B (10 min). The flow rate was set at 200 μL/min and the UV was monitored at 214 nm.

The Orbitrap Exploris 240 MS system was operated in the positive ion mode. Tandem MS/MS analyses were performed for the identification of peptide in data dependent mode. Full MS scan data acquired within a 200-2000 m/z scan range, 60,000 resolution over 100ms injection time, followed by 5 sequential MS/MS scan with orbitrap resolution target of 15000. A minimum intensity threshold was set to 1000 with a custom dynamic exclusion filter applied. Charge states were filtered to include charges of 2-8 and the number of dependent scans was set to 5. A 2 m/z isolation window was applied for the ddMS scan with HCD collision energies set to 20, 25 and 30% over 200 ms injection time. MS2 data acquired in profile mode. The MS2 AGC target was set at 100% whereas full scan AGC target was set at 300%. Byos software (version 5.0-88 (2022.12), Protein Metrics, CA, USA) was used to processing of peptide fragments using the following parameters: Precursor Mass Tolerance set at 20 ppm, Fragment Mass Tolerance 1 and 2 set at 20 ppm, Cleavage Site(s) set as RK (trypsin) and WFLY (chymotrypsin), Missed Cleavages set at 2, Cleavage Side set as C-terminal and Fragmentation type set as QTOF/HCD. The post translation modifications (PTMs) screened for were methylation, oxidation, deamidation and pyroglutamate formation.

Verification of anti-IL8 WT and mutant variant identity by peptide fragmentations. Trypsin or chymotrypsin digest of this peptide following the same methodology showed coverage of this missing peptide, ensuring full identity verification. For post-translational modifications (PTMs), the % detection was relative to only peptides with expected full enzyme cleavage. PTMs with relative detection were noted. *HC: Heavy chain; LC: Light chain; mwt: molecular weight; PTM: post-translational modification*

| **Molecule** | **LC coverage (%)** | **HC coverage (%)** | **LC mwt (Da)** | **HC mwt (Da)** | **LC PTMs** | **HC PTMs** |
| --- | --- | --- | --- | --- | --- | --- |
| WT | 97.66 | 96.66 | 23433.83 | 49204.09 | M4 oxidation (0.4%) | M81 oxidation (0.2%),  N317 deamidation (0.6%), M254 oxidation (4.2%), N363 deamidation (0.4%), M430 oxidation (1.8%) |
| D17N | 97.66 | 97.44 | 23432.85 | 49204.09 | M4 oxidation (0.3%) | M81 oxidation (0.2%), M254 oxidation (4.5%), N317 deamidation (0.1%), N363 deamidation (0.4%), M430 oxidation (2.1%) |
| D70N | 99.07 | 98.22 | 23432.85 | 49204.09 | M4 oxidation (0.3%), p*ossible N70 deamidation but not confirmed due to poor fragmentation (see map coverage below Figure S3)* | M81 oxidation (0.2%), M254 oxidation (4.8%), N288 deamidation (0.1%), N317 deamidation (0.3%), N363 deamidation (0.2%), N436 deamidation (1.42%) |
| K42E | 97.66 | 98.22 | 23434.77 | 49204.09 | M4 oxidation (0.3%) | M81 oxidation (1.12%), N317 deamidation (0.4%), M430 oxidation (1.9%), N436 deamidation (3.3%) |
| V5Q | 97.66 | 98.22 | 23433.83 | 49233.09 | None | N363 deamidation (0.2%) |
| W32Q | 97.66 | 79.73* | 23433.83 | 49146.01 | None | S methylation (100%), N317 deamidation (2.5%) |
| D28N | 97.66 | 98.22 | 23432.85 | 49204.09 | N28 deamidation (12.3%) | N317 deamidation (0.4%), N363 deamidation (0.2%), N436 deamidation (1%) |
| D56N | 97.66 | 98.22 | 23432.85 | 49204.09 | N56 deamidation (1.85%) | N436 deamidation (0.8%) |
| R53G | 97.66 | 98.22 | 23334.7 | 49204.09 | M4 oxidation (1%) | M430 oxidation (1.6%) |

***Analysis of Monomeric Purity by Analytical Size Exclusion Chromatography (aSEC)***

Samples were injected onto a TSKgel Super SW3000, 4.6 x 300 mm (TOSOH Bioscience, United States) column on an Agilent 1260 series HPLC, with 0.1M sodium phosphate containing 400 mM NaCl (pH 6.8) as the mobile phase. All samples were analysed at 5 mg/mL at a 0.2 mL/min flow rate, and detected at 280 nm. The OpenLab CDS Data Analysis software (version 2.6, Agilent, California, US) was used to process and integrate the chromatograms. Areas under the chromatographic peaks were integrated to quantify the monomeric mAb, and high and low molecular weight species. The target monomeric purity of ≥95% was met by all WT and mutant anti-IL8 molecules and aSEC was used to monitor physicochemical stability, by monitoring changes in chromatogram peak retention times and profiles for each molecule. Analysis of the expressed anti-IL8 mutants showed retention times comparable to the anti-IL8 WT IgG1 (~27.5 minutes), except for the D70N mutant, which had a consistent reduced retention time of ~26.6 minutes suggesting a slight increase in molecular size.

Monomeric purity of all anti-IL8 molecules (N=3).

| Mab | RT (min) | Peak Width (min) | %HMW species | %Monomer | % LMW species |
| --- | --- | --- | --- | --- | --- |
| *WT* | 27.5 (±0.5) | 0.53 (±0.05) | 1.3 (±0.2) | 97.1 (±0.5) | 1.6 (±0.5) |
| *D17N (FWL)* | 27.4 (±0.3) | 0.51 (±0.05) | 1.2 (±0.1) | 98.0 (±0.5) | 0.9 (±0.5) |
| *D70N (FWL)* | 26.6 (±0.3) | 0.75 (±0.03) | 1.5 (±0.2) | 98.0 (±0.4) | 0.6 (±0.3) |
| *K42E (FWL)* | 27.3 (±0.3) | 0.51 (±0.05) | 1.4 (±0.2) | 97.2 (±0.6) | 1.4 (±0.5) |
| *V5Q (FWH)* | 27.5 (±0.5) | 0.53 (±0.06) | 1.2 (±0.3) | 97.4 (±0.7) | 1.4 (±0.5) |
| *W32Q (CDRH2)* | 27.5 (±0.6) | 0.51 (±0.05) | 2.1 (±0.6) | 96.8 (±1.1) | 1.1 (±0.5) |
| *D28N (CDRL1)* | 27.6 (±0.5) | 0.55 (±0.1) | 1.8 (±0.3) | 96.5 (±0.8) | 1.7 (±0.8) |
| *D56N (CDRL2)* | 27.5 (±0.5) | 0.53 (±0.05) | 1.4 (±0.6) | 97.0 (±1.1) | 1.7 (±0.5) |
| *R53G (CDRL1)* | 27.5 (±0.5) | 0.53 (±0.05) | 1.5 (±0.4) | 97.2 (±0.7) | 1.2 (±0.4) |

***Hydrophobic interaction chromatography***

***
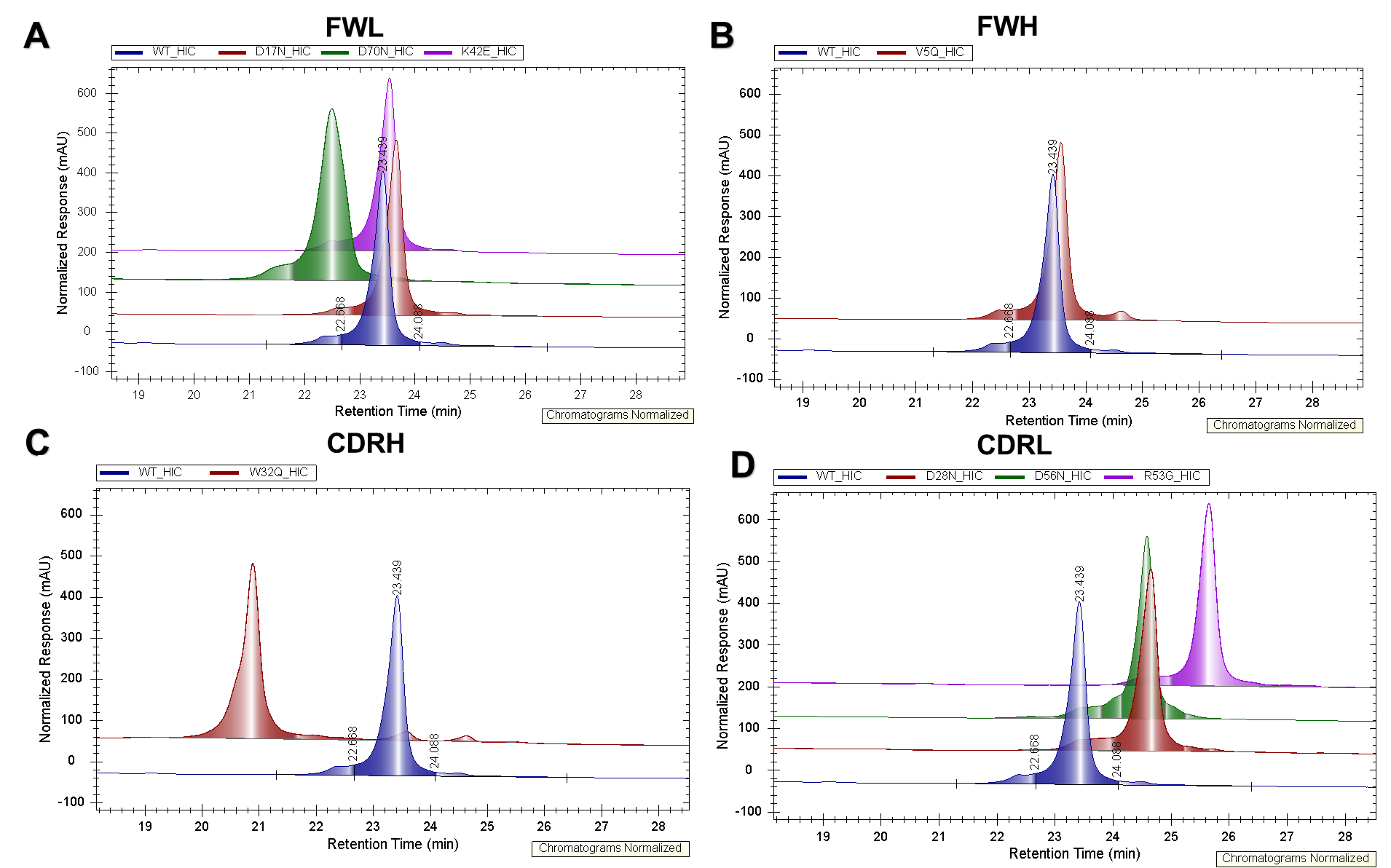
***

Hydrophobic interaction chromatography chromatograms for anti-IL8 mutants. For all chromatograms, the WT (blue) retention time was observed at ~23.5 minutes. For FWL mutants (A), only D70N (green) had a shift in retention time and an increase in peak breadth. A slight increase in retention time was seen for FWH (B) V5Q mutant (red), and a large reduction in retention time for the CDRH (C) W32Q mutant (red), demonstrating the impact of size of the original hydrophobic patch targeted. For all CDRL mutants (D), an increased retention time was observed relative to WT, particularly for R53G (pink) where there was a predicted increase in neighbouring hydrophobic patch size from this disruption of a positive patch.

**Binding analysis**

We used a Biacore 8K+ surface plasmon resonance (Cytiva, Danaher, USA) to compare the association and dissociation rates, and affinity for to IL-8 carrier-free antigen (R&D systems, USA) between the WT and mutant panel. IL-8 (0.5 µg/mL) was immobilised onto one flow cell of a Biacore CM3 dextran chip (Cytiva, Danaher, USA). The experiment consisted of ten start-up cycles, followed by ten antibody injections at a flow rate of 30 μL/min and a temperature of 25 °C. The contact time was 240 seconds, and dissociation was monitored over 900 seconds after injection. All antibodies (0.31-20 µg/mL) were formulated in phosphate-buffered saline containing 0.05% Tween 20, with the same running buffer composition. Surfaces were regenerated between measurements using 10 mM glycine (pH 1.5) and 3 M guanidine. The data were analyzed using Biacore Insight Evaluation software (version 4.0.8.20368, Cytiva, USA) with a 1:1 Langmuir binding model.

To determine the apparent dissociation (ka) and dissociation rate constants (kd). The $\frac{k_{d}}{k_{a}}$ ratio was used to determine the equilibrium dissociation constant (KD).

The impact of introducing single-point mutations on the ligand binding affinity of anti-IL8 mutant variants was measured by SPR. The mean binding affinity across all mutant variants was equivalent to the anti-IL8 WT (3.92 nM), except for the W32Q mutant (CDRH), which had no binding affinity for the target antigen.

Biacore analysis of binding kinetics. Wild-type and mutant anti-IL8 binding to an IL-8 antigen was assessed with SPR. Data in the table includes the binding on-rate (k_a_), the binding off-rate (k_d_) and the equilibrium dissociation constant (KD), as well as the maximum response (R_max_) and goodness of fit (Chi-squared) of the 1:1 binding model. All framework mutants and CDRL mutants showed no significant change in affinity relative to the anti-IL8 WT. The W to Q single point mutation in the CDRH2 domain knocked out all binding affinity to IL8 antigen. FWL: light chain framework region; FWH: heavy chain framework region; CDRH2: heavy chain complementarity-determining region 2; CDRL1: light chain complementarity-determining region 1; CDRL2: light chain complementarity-determining region 2 (N=2).

| Molecule | 1:1 binding kinetics | | | | Kinetics (Χ^2^) |
| --- | --- | --- | --- | --- | --- |
|  | **k_a_ x10^5^**  (M^-1^s^-1^) | **k_d_ x10^-4^** (s^-1^) | **KD** (nM) | **R_max_** (RU) |  |
| *WT* | 2.53 (±0.13) | 9.90(±0.04) | 3.92(±0.18) | 23.35(±0.78) | 0.94(±0.01) |
| *D17N (FWL)* | 2.89(±0.01) | 9.78(±0.03) | 3.39(±0.01) | 27.75(±0.07) | 1.48(±0.07) |
| *D70N (FWL)* | 2.57(±0.13) | 0.102(±0.14) | 3.98(±0.26) | 20.9(±0.71) | 0.038(±0.04) |
| *K42E (FWL)* | 2.49(±0.04) | 9.54(±0.06) | 3.84(±0.08) | 19.65(±0.07) | 0.72(±0.01) |
| *V5Q*  *(FWH)* | 2.13(±0.01) | 9.82(±0.03) | 4.62(±0.03) | 24.55(±0.07) | 2.01(±0.08) |
| *W32Q (CDRH2)* | 28.6(±6.01) | 0.37(±0.05) | 0.01(±0.02) | 0.45(±0.07) | 0.06(±0.00) |
| *D28N (CDRL1)* | 2.60(±0.01) | 11.00 (±0.01) | 4.24(±0.06) | 26.1(±0.14) | 1.19(±0.07) |
| *D56N (CDRL2)* | 3.12(±0.01) | 10.5(±0.01) | 3.38(±0.11) | 29.1(±0.42) | 1.74(±0.04) |
| *R53G* (CDRL2) | 3.07(±0.01) | 11.5(±1.63) | 4.17(±0.16) | 13.5(±0.28) | 1.96(±0.04) |

***Differential Scanning Fluorimetry (DSF)***


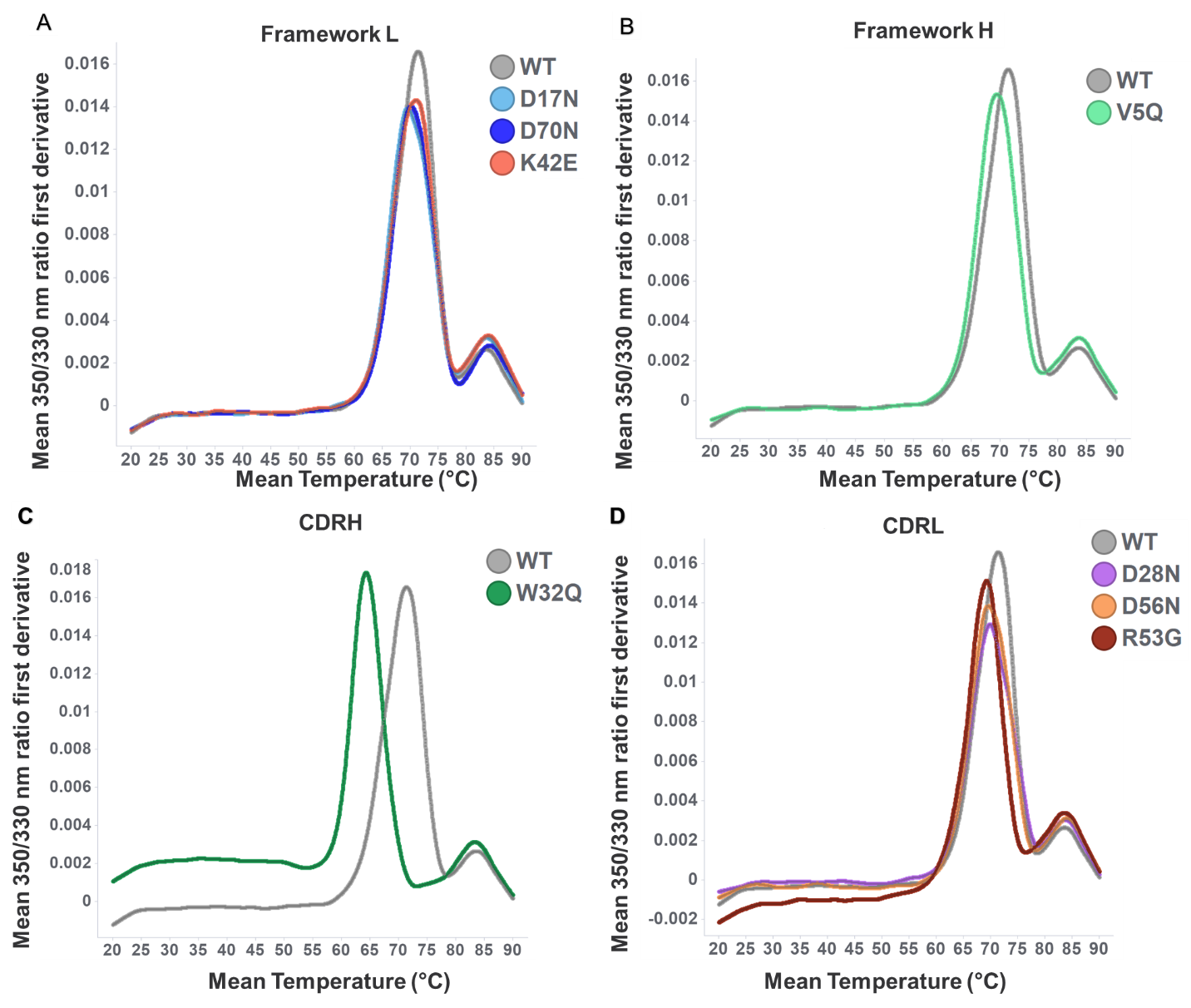


Thermal unfolding profiles for all generated mutant molecules categorised by mutation site. 350nm/330nm ratio was obtained from differential scanning fluorimetry experiments for all expressed mutants and anti-IL8 WT at 150 mg/mL. To identify melting temperature peaks, the first derivative was calculated. The onset of unfolding (T_onset_) was identified at the inflection point of the first peak and was approximately 62°C for most mutants.

Thermal parameters derived from differential scanning fluorimetry of the WT and mutant anti-IL8 panel. Unfolding onset temperatures (T_onset_) were comparable for all anti-IL8 mutants (~63°C), except for W32Q (CDRH) and R53G (CDRL), which had lower T_onsets_. Reduced thermal stability for W32Q was supported with reduced T_m1_ and T_agg_ values compared to WT. The distinction of lower thermal stability for R53G was weaker with a large deviation for T_agg_. All anti-IL8 samples were measured at 150 mg/mL. Only T_m1_ and T_m3_ peaks were detected for all molecules. FWL: light chain framework region; FWH: heavy chain framework region; CDRH2: heavy chain complementarity-determining region 2; CDRL1: light chain complementarity-determining region 1; CDRL2: light chain complementarity-determining region 2 (N=2).N=2 biological replicates.

| Molecule | T_onset_ (°C) | T_m1_ (°C) | T_m3_ (°C) | T_agg_ (°C) |
| --- | --- | --- | --- | --- |
| *WT* | 62.85(±0.34) | 71.28(±0.17) | 83.71(±0.08) | 71.31(±2.40) |
| *D17N*  *(FWL)* | 63.19(±0.08) | 69.76(±0.13) | 83.61(±0.01) | 70.90(±0.53) |
| *D70N*  *(FWL)* | 63.32(±0.27) | 70.17(±0.04) | 84.25(±0.06) | 73.04(±4.12) |
| *K42E*  *(FWL)* | 63.18(±0.07) | 70.85(±0.11) | 83.97(±0.00) | 70.57(±1.14) |
| *V5Q*  *(FWH)* | 62.74(±0.28) | 69.42(±0.07) | 83.55(±0.06) | 69.59(±0.79) |
| *W32Q*  *(CDRH2)* | 60.08(±0.13) | 64.30(±0.01) | 83.27(±0.01 | 64.80(±0.96) |
| *D28N*  *(CDRL1)* | 62.56(±0.34) | 69.86(±0.26) | 83.65(±0.23) | 71.87(±1.59) |
| *D56N*  *(CDRL2)* | 62.51(±0.54) | 69.80(±0.81) | 83.88(±0.47) | 70.57(±2.39) |
| *R53G*  *(CDRL1)* | 58.63(±0.04) | 69.09(±0.20) | 83.52(±0.05) | 68.34(±10.46) |

***Dynamic light scattering (DLS)*** ***measurements***

***Concentration-dependent diffusion coefficient data*** 
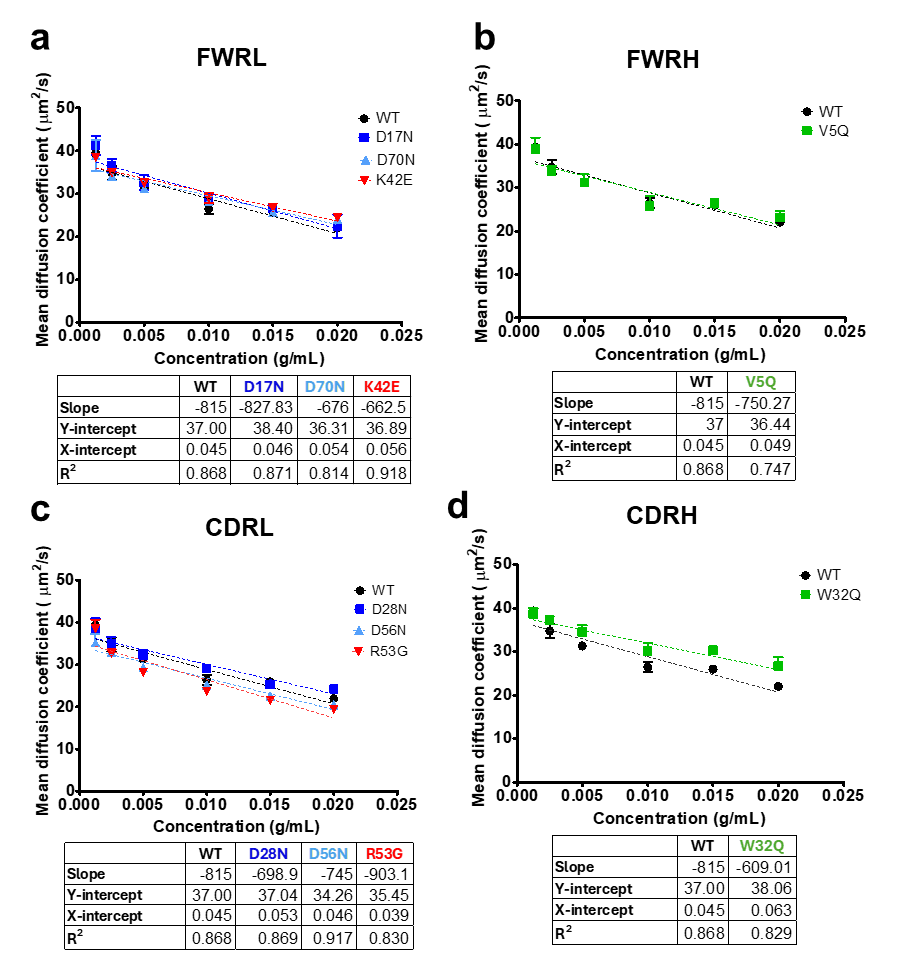


**Diffusion coefficients for each expressed anti-IL8 mutant and WT over a dilute concentration range (1-20 mg/mL), fitted with linear regression. Goodness of fit R-squared values are reported along with linear equations used to calculate self-interaction kD values by dividing the slope over the Y intercept Error bars for each measurement represent standard deviation.**

**Mean hydrodynamic diameters (Z-ave) and polydispersity indices for anti-IL8 WT and mutant panel over a dilute concentration range (1-20 mg/mL), derived from dynamic light scattering measurements. Error bars represent standard deviations, N=3.**

Dynamic light scattering measurements were also used to interpret the second virial coefficients (B_22_) of each anti-IL8 molecule^[[1]](#endnote-2)^:

$\frac{Kc}{R}=\frac{1}{MwP_{0}}+2B_{22}c$ (**S1**)

Where K is the optical constant, c is the concentration (g/mL), R is the Raleigh ratio of scattered light to incident light, Mw is the molecular weight of the protein (g/mol), P_0_ is the angular scattering dependence (assumed equal to 1).

Kc/R values per molecule were exported from the Stunner analysis software (v8.1.0.244, Unchained Labs, CA, US) and plotted over the concentration range tested (1-20 mg/mL). The slope of the linear regression was divided by 2 to generate B_22_ values.

**
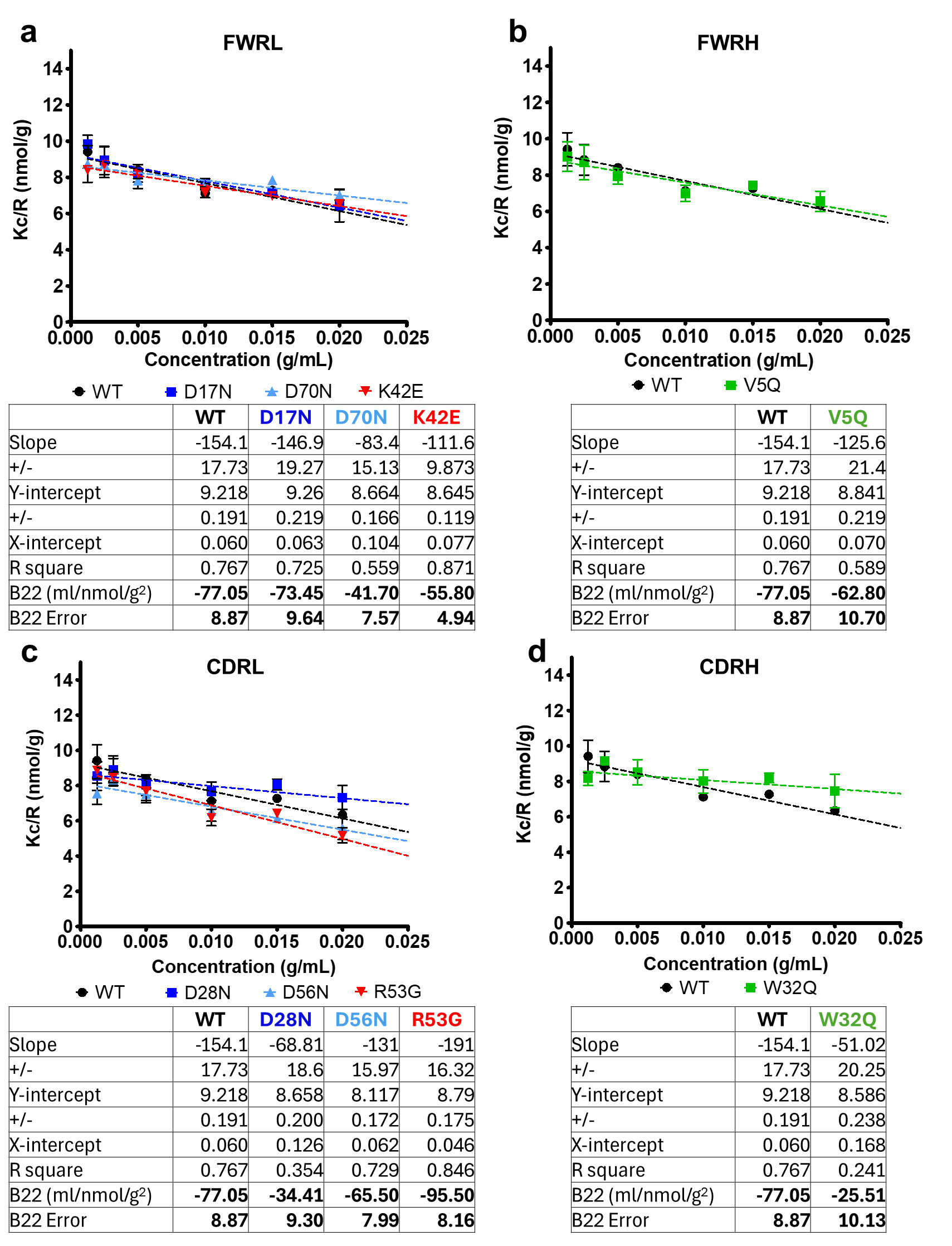
**

**The second virial coefficient, B_22_ (mL/nmol/g^2^), was derived from dynamic light scattering using the slope of Kc/R over the 1-20 mg/mL concentration range, where K is the optical constant, c is the concentration (g/mL), and R is the Raleigh ratio. Error bars represent standard deviations of measurements (N=3). Linear fits are plotted, with the equation parameters as well as R^2^ goodness-of-fit values.**

***Viscosity modelling***

**An exponential growth model (Equation 6) was used to fit concentration-viscosity profiles of anti-IL8 WT and mutant molecules. Equation parameters and goodness of fit R^2^ are reported.**

| **Parameter** | **WT** | **D17N** | **D70N** | **K42E** | **V5Q** | **D28N** | **D56N** | **R53G** | **W32Q** |
| --- | --- | --- | --- | --- | --- | --- | --- | --- | --- |
| **Y0** | 0.848 | 0.031 | 1.14 | 0.027 | 0.779 | 0.797 | 0.852 | 1.03 | 1.49 |
| **k** | 0.022 | 0.063 | 0.015 | 0.066 | 0.032 | 0.026 | 0.024 | 0.03 | 0.012 |
| **R^2^** | 0.975 | 0.936 | 0.998 | 0.984 | 0.984 | 0.939 | 0.835 | 0.824 | 0.551 |

1. Ma et al., “Determination of the Second Virial Coefficient of Bovine Serum Albumin under Varying pH and Ionic Strength by Composition-Gradient Multi-Angle Static Light Scattering.” [↑](#endnote-ref-2)
